# CXCL13 in Cerebrospinal Fluid: Clinical Value in a Large Cross-Sectional Study

**DOI:** 10.3390/ijms25010425

**Published:** 2023-12-28

**Authors:** Deborah Katharina Erhart, Veronika Klose, Tatjana Schäper, Hayrettin Tumani, Makbule Senel

**Affiliations:** 1 Department of Neurology, University Hospital of Ulm, Oberer Eselsberg 45, 89081 Ulm, Germany; deborah.erhart@uni-ulm.de (D.K.E.); tatjana.schaeper@rku.de (T.S.); makbule.senel@uni-ulm.de (M.S.); 2 German Center for Neurodegenerative Diseases (DZNE)—Ulm, Oberer Eselsberg 45, 89081 Ulm, Germany; veronika.klose@dzne.de

**Keywords:** CXCL13, CNS-inflammatory diseases, cerebrospinal fluid, neuroborreliosis

## Abstract

C-X-C-motif chemokine ligand 13 (CXCL13) in cerebrospinal fluid (CSF) is increasingly used in clinical routines, although its diagnostic specificity and divergent cut-off values have been defined so far mainly for neuroborreliosis. Our aim was to evaluate the value of CSF-CXCL13 as a diagnostic and treatment response marker and its role as an activity marker in a larger disease spectrum, including neuroborreliosis and other neuroinflammatory and malignant CNS-disorders. Patients who received a diagnostic lumbar puncture (LP) (*n* = 1234) between July 2009 and January 2023 were included in our retrospective cross-sectional study. The diagnostic performance of CSF-CXCL13 for acute neuroborreliosis was highest at a cut-off of 428.92 pg/mL (sensitivity: 92.1%; specificity: 96.5%). In addition, CXCL13 levels in CSF were significantly elevated in multiple sclerosis with clinical (*p* = 0.001) and radiographic disease activity (*p* < 0.001). The clinical utility of CSF-CXCL13 appears to be multifaceted. CSF-CXCL13 is significantly elevated in patients with neuroborreliosis and shows a rapid and sharp decline with antibiotic therapy, but it is not specific for this disease and is also highly elevated in less common subacute neuroinfectious diseases, such as neurosyphilis and cryptococcal meningitis or in primary/secondary B-cell lymphoma.

## 1. Introduction

Chemokine C-X-C-motif chemokine ligand 13 (CXCL13) has gained attention for its essential role in germinal center organization and compartmentalization in secondary lymphoid tissue [1]. The chemoattractant is produced by mononuclear antigen-presenting cells (monocytes, macrophages, follicular dendritic cells, and natural killer cells) and directs B-lymphocytes and B-helper follicular T-lymphocytes, all of which express the specific C-X-C chemokine receptor type 5 (CXCR5+), into germinal centers of B cell follicles in secondary or tertiary lymphoid organs [2,3,4,5,6]. Interactions with Toll-like receptor 2 (TLR2) on monocytes mediated by the special surface lipoproteins of spirochetes (outer surface protein A (OspA) of Borrelia burgdorferi, N-palmitoyl-S-(bis(palitoyloxy)propyl)cystein (Pam3C) of Treponema pallidum) serve as triggers for CXCL13 production, in contrast to pneumococci [7]. CSF-CXCL13 is highly elevated in early neuroborreliosis before borrelia-specific antibody production is detectable [8,9,10,11,12]. In neurological inflammatory diseases, the value of CSF-CXCL13 correlates with the level of CSF pleocytosis, CSF/serum albumin ratio, level of intrathecal immunoglobulin synthesis, CSF-specific oligoclonal IgG bands, B cells, plasmablasts, CXCR5+ T cells, and monocytes [4,13,14,15,16]. The marked elevation of CSF-CXCL13 has also been reported in neurosyphilis, primary and secondary CNS lymphoma, autoimmune encephalitis, multiple sclerosis, and cryptococcosis with CNS involvement [17,18,19,20].

As a result, there were discussions concerning the specificity of CSF-CXCL13 [21]. For this purpose, specific cut-off values (18.9–1726 pg/mL) were established as an attempt to distinguish Lyme neuroborreliosis (LNB) from differential diagnoses and other neurological diseases with high sensitivity and specificity [8,9,10,13,21,22,23,24,25,26,27,28,29,30,31,32,33,34,35,36,37,38]. Due to the wide range, no official cut-off has yet been determined. In addition, CSF-CXCL13 serves as a treatment response marker in neuroborreliosis that decreases most strongly and rapidly during therapy, independent of the decrease in the CSF/serum albumin ratio or total cell count [9,39]. Studies regarding the prognostic value of CXCL13, its dependence on the severity of the disease, and the disease course (uncomplicated vs. complicated course) have so far only been carried out in small patient cohorts (*n* ≤ 26) and showed a tendency towards higher CSF-CXCL13 levels in complicated courses [40,41].

In the meantime, there is a large number of studies on CXCL13, mainly in neuroborreliosis but also to a lesser extent in various other infectious, autoimmune, and malignant neurological diseases with different study populations, diseases, and detected cut-offs for CSF-CXCL13 [8,10,13,14,15,16,18,19,20,26,27,32,37,40,42,43]. The role of CXCL13 in various diseases has yet to be apparent due to the differing study situations. CSF-CXCL13 has therefore yet to find its way into routine diagnostics outside of large tertiary centers and university hospitals. Therefore, we have set the target of retrospectively investigating levels of CXCL13 in the CSF of a large real-life population over an extended period (July 2009–January 2023). Our retrospective cross-sectional study aimed to investigate the diagnostic capacity, treatment response, and value for the severity of CSF-CXCL13 in different neurological diseases to provide an overview, especially for clinicians, of what an increase in CXCL13 may indicate. The purpose of this study was to (1) present an overview of different neurological diseases with measurements of CSF-CXCL13, (2) correlate CSF-CXCL13 with other CSF measures, such as the leucocyte and albumin ratio (QAlb), (3) examine the role of CSF-CXCL13 as a treatment response and activity marker in neuroborreliosis and other inflammatory CNS diseases, including multiple sclerosis, (4) determine the role of CSF-CXCL13 as a differential diagnostic parameter of neuroborreliosis and various neurological diseases, and (5) investigate the potential role of CSF-CXCL13 as a severity marker in different disease courses (complicated vs. uncomplicated) in the neuroinfectious disease group.

## 2. Results

### 2.1. CSF-CXCL13 in Different Neurological Diseases

**Figure 1:**
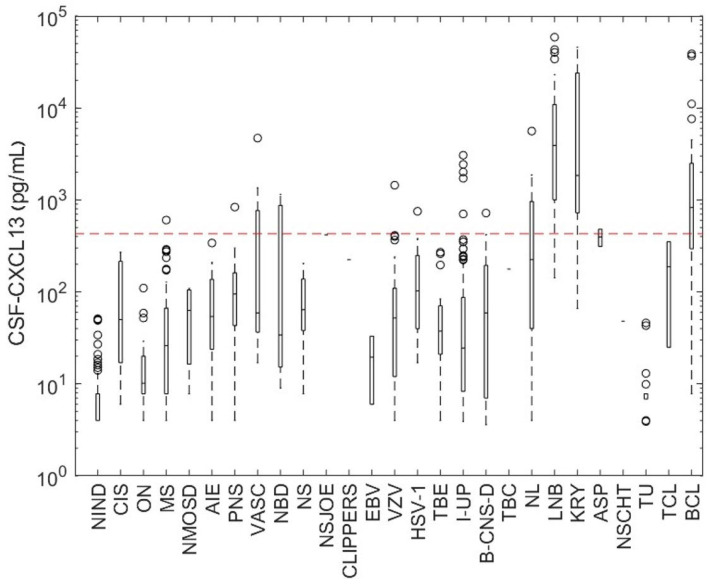
CSF-CXCL13 in various neurological disorders. The selected patients from the Biobank are represented based on the level of CXCL13 and the respective disease. CXCL13 concentrations in the different neurological diseases are illustrated as boxplots. Their bars represent interquartile ranges (IQR; 25–75% percentile), horizontal lines indicate medians, dotted lines represent the upper and lower whiskers (+/−1.5 × IQR), and open circles represent outliers. The red dashed line represents a reference line for the CXCL13 cut-off of 428.92 pg/mL calculated with receiver operating characteristic (ROC) curve analysis to distinguish neuroborreliosis from other neuroinflammatory diseases with the highest sensitivity and specificity. NIND: non-inflammatory neurological diseases, CIS: clinically isolated syndrome, ON: optic neuritis, MS: multiple sclerosis, NMOSD: neuromyelitis optica spectrum disease, AIE: autoimmune (limbic) encephalitis, PNS: paraneoplastic syndrome, VASC: CNS-vasculitis, NBD: Neuro-Behçet’s disease, NS: neurosarcoidosis, NSJOE: Sjögren’s syndrome (neurological), CLIPPERS: chronic lymphocytic inflammation with pontine perivascular enhancement responsive to steroids, EBV: Epstein–Barr virus, VZV: varicella-zoster virus, HSV-1: herpes simplex virus-1, TBE: tick-borne meningoencephalitis, I-UP: neuroinfectious diseases of unknown pathogens, B-CNS-D: bacterial meningitis, TBC: tuberculosis, NL: neurosyphilis, LNB: Lyme neuroborreliosis, KRY: cryptococcosis, ASP: aspergillosis, NSCHT: neuroschistosomiasis, TU: tumor of the CNS, TCL: T-cell lymphoma, BCL: B-cell CNS-lymphoma.

First, we would like to give an overview of the diversity of diseases in which CXCL13 was determined in the CSF (Figure 1). The investigations included various disease patterns of different etiologies: infectious inflammatory (viruses, bacteria, fungi, parasites) and autoimmune (inflammatory) diseases, primary or secondary CNS neoplasms, rheumatologic and granulomatous diseases with CNS involvement, and non-inflammatory controls. Finally, in some cases (*n* = 101), no pathogen could be identified (=neuroinfectious diseases of unknown pathogens (I-UPs)). The highest CSF-CXCL13 levels were found in neuroborreliosis (median 3920 pg/mL; range: 142–23,055 pg/mL), cryptococcosis with CNS-involvement (median 1843.5 pg/mL; range: 66–45,609 pg/mL), and B-cell lymphoma (primary or secondary CNS-involvement; median 826.3 pg/mL; range: 7.8–4500 pg/mL). In Dunn’s post hoc multiple comparisons, these three diseases did not differ significantly regarding CSF-CXCL13 (*p* > 0.05). CSF-CXCL13 levels in optic neuritis, clinically isolated syndrome, neuromyelitis optica spectrum diseases, EBV-meningitis, and Neuro-Behçet’s disease (NBD) were not significantly different from those in the control group (*p* > 0.05). Moreover, the level of CSF-CXCL13 did not significantly (*p* > 0.05) differentiate MS, neuroinfectious diseases of unknown pathogens (I-UPs), TBE, VZV infections, limbic encephalitis, neurosarcoidosis, paraneoplastic syndrome (other than limbic encephalitis), and bacterial meningitis from each other.

### 2.2. Patient and Demographic Characteristics

In our further analyses, we included paired CSF/serum samples from the selected 677 patients (plus another 105 samples for follow-up comparisons) (groups and related diseases in Table S1). The age, leucocyte cell count, total protein, and QAlb were not normally distributed in all four groups (Table 1). The groups differed significantly (*p* < 0.001) in gender. In the NID group, the proportion of women was lowest (36.1%) compared to that in the AIND group (59.2%). Similarly, the groups differed significantly (*p* < 0.001) concerning age. The median age was highest in the NPL group at 66 (range: 34–86) and lowest in the AIND group (median 45; range: 4–88). No significant difference in gender (*p* = 0.384) and no correlation or significance in age (r = 0.079 and *p* = 0.156) was found for CSF-CXCL13 in the control group (NIND) (Figure S1). An overview of patient and demographic characteristics and the routine CSF findings is provided in Table 1 and Table S2. The median leucocyte cell count (101/µL; range: 6–480/µL), total protein (957 mg/L; range: 179–3268 mg/L), QAlb (12.5; range: 2.4–34.6), proportion of positive intrathecal IgM synthesis (32.2%), and CXCL13 (76 pg/mL; range: 4–1466 pg/mL) were highest in the NID group. The percentage of CSF-specific oligoclonal IgG bands (pattern 2 or 3) was highest in the AIND group (78.4%).

### 2.3. Correlation between CSF-CXCL13 and Other Routine CSF Parameters

We performed correlation analyses using Spearman’s coefficient “r” between CSF-CXCL13 and various parameters of CSF routine diagnostics, i.e., the leucocyte cell count, differential cytology (absolute cell count for monocytes, lymphocytes, plasma cells), and QAlb in the four groups (Table 2). Regarding the absolute leucocyte and lymphocyte counts, we found a moderate-to-strong correlation (maximum r = 0.748 (lymphocytes in NPL); minimum r = 0.495 (lymphocytes in AIND)) in the AIND and NPL group. In the NID group, only a weak and significant correlation with CSF-CXCL13 was observed regarding leucocyte and lymphocyte counts (r = 0.132 and r = 0.139, respectively). For the NIND group, no significant correlation with the CSF-CXCL13 level was found for leucocyte, lymphocyte, and monocyte counts. Regarding QAlb, there was a significant correlation with CSF-CXCL13 in all groups, except for in the NIND group despite a predominantly intrathecal origin of CXCL13 (Table S3). The strongest correlation was for the NPL group (r = 0.674). In the NID group, CSF-CXCL13 was significantly higher (*p* < 0.001) when CSF-specific oligoclonal IgG bands could be detected (Figure S2). This was not found in the AIND and NPL groups (*p* = 0.273 and *p* = 0.307, respectively). In the case of intrathecal IgM synthesis, CSF-CXCL13 was considerably higher in all three disease groups and most significantly for NID (*p* < 0.001) (Figure S2).

### 2.4. CSF-CXCL13 as a Treatment Response Marker in LNB and Other Infectious Neurological Diseases

We found a strong and significant correlation between CSF-CXCL13 and routine diagnostic CSF parameters in neuroborreliosis (Table S4). The strongest correlation was obtained for plasma cells (r = 0.508) and lymphocytes (r = 0.471). In addition, CSF-CXCL13 values were higher when oligoclonal IgG bands (*p* = 0.022) or intrathecal IgM synthesis (*p* < 0.001) were present in CSF. The median time from symptom onset to the first lumbar puncture was seven days (range: 1–45 days). The level of CSF-CXCL13 did not correlate significantly with symptom duration prior to the first lumbar puncture (*p* = 0.451).

**Figure 2:**
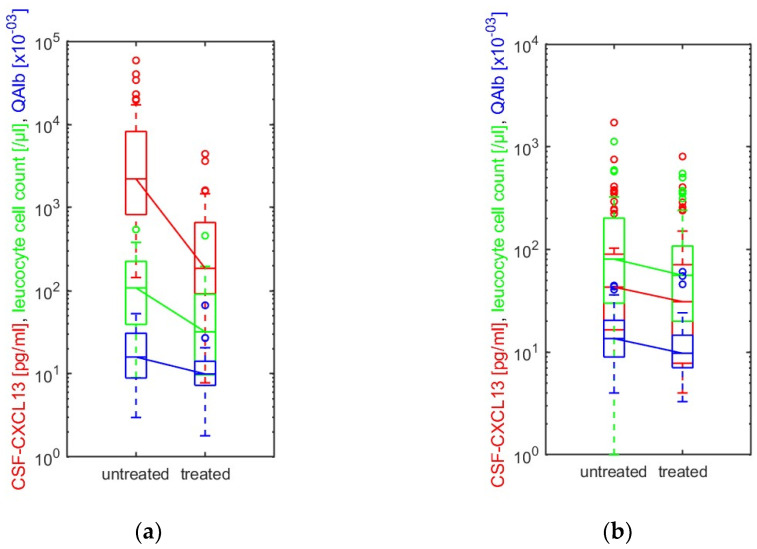
(**a**) Thirty-eight patients with neuroborreliosis and (**b**) 47 patients with viral diseases (known pathogen) and neuroinfectious diseases of unknown pathogens received another lumbar puncture after starting antibiotic or antiviral therapy. The CSF-CXCL13 level (red), the leucocyte cell count (green), and QAlb (blue) in (**a**) neuroborreliosis and (**b**) viral (known pathogen) and neuroinfectious diseases of unknown pathogens are illustrated before (“untreated”) and after (“treated”) anti-infective treatment. The CXCL13 value (pg/mL; red), leucocyte cell count (/µL; green), and QAlb (CSF/serum albumin ratio ×10^−03^; blue) in CSF before (“untreated”) and after (“treated”) the initiation of therapy are shown as boxplots with medians (horizontal lines), interquartile ranges (bars), whiskers (dotted lines), and outliers (open circles). A line connects the corresponding medians. We used the Wilcoxon signed-rank test to compare CXCL13 in CSF, leucocyte cell counts, and QAlb before and after treatment. *p*-values <0.05 were considered statistically significant. (**a**) CSF-CXCL13 (red): *p* < 0.001; leucocyte count (green): *p* < 0.001; QAlb (blue): *p* < 0.001; (**b**) CSF-CXCL13 (red): *p* = 0.046; leucocyte count (green): *p* = 0.044; QAlb (blue): *p* = 0.033. QAlb: CSF-albumin/serum-albumin (albumin ratio).

Thirty-eight neuroborreliosis patients received a second spinal tap within a median of ten days (range: 1–25 days) related to the first lumbar puncture after the initiation of antibiotic therapy with ceftriaxone at 2 g/day or doxycycline at 200 mg/day (two patients, partly due to allergy to cephalosporins). A third lumbar puncture was performed for nine patients (median 14 days; range: 6–20 days). A comparison of different baseline CSF parameters and CXCL13 at the first (“untreated”) and last (“treated”) lumbar puncture was performed (Figure 2a). After therapy initiation, CSF-CXCL13 dropped significantly (*p* < 0.001), together with the leucocyte count (*p* < 0.001) and QAlb (*p* < 0.001). In viral CNS-inflammatory diseases or neuroinfectious diseases of unknown pathogens (I-UPs), another spinal tap was also performed for 47 patients with a median of seven days (range: 1–15 days) after the start of anti-infective therapy. Most patients initially received antibiotic therapy with ceftriaxone and/or ampicillin/sulbactam and acyclovir until further results were obtained. Here, the decrease in the leucocyte count (*p* = 0.044), QAlb (*p* = 0.033), and CXCL13 (*p* = 0.046) was only slightly significant as shown in Figure 2b. However, it was evident that the decrease in CSF-CXCL13 in this group was not as significant as that in the neuroborreliosis patients.

### 2.5. CSF-CXCL13 as an Activity Parameter in Multiple Sclerosis and Related Diseases

**Figure 3:**
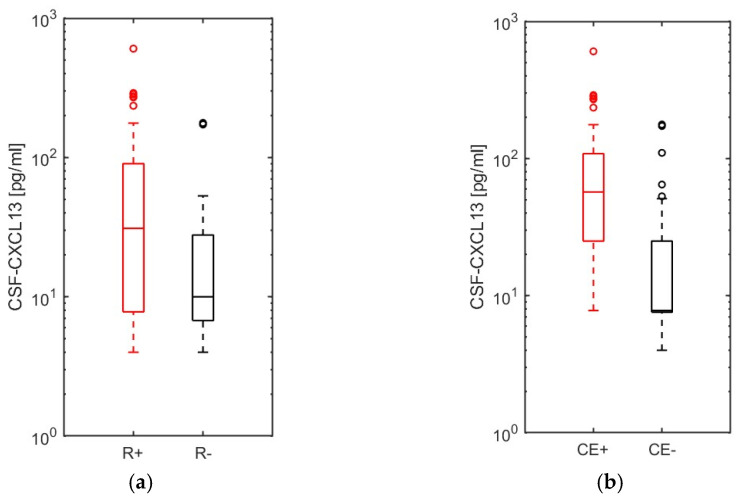
CSF-CXCL13 as an activity parameter in multiple sclerosis and related diseases with (**a**) clinical and (**b**) radiographic relapse symptoms. The 98 patients with multiple sclerosis (MS), optic neuritis (ON), clinically isolated syndrome (CIS), and neuromyelitis optica spectrum disease (NMOSD) were categorized according to clinical relapse symptoms (R+/−) and contrast-enhancing (CE+/−) T1 lesions on magnetic resonance imaging (MRI) by location. CXCL13 levels in the CSF were compared between MS/ON/CIS/NMOSD patients with and without (**a**) clinical or (**b**) radiographic relapse symptoms. Data are represented as boxplots with interquartile range (bars), whiskers (dotted lines), medians (horizontal lines), and outliers (open circles). The Mann-–Whitney U test was used to compare the two groups in (**a**,**b**). *p*-values <0.05 were considered statistically significant. (**a**) *p* = 0.001; (**b**) *p* < 0.001.

Multiple sclerosis, isolated optic neuritis, CIS, and NMOSD patients (*n* = 98) were clinically (+/− relapse symptoms) and radiographically (+/− new gadolinium-enhancing T1 lesion) classified (Figure S3). Forty-three patients with relapse symptoms had at least one gadolinium-enhancing T1 lesion, supra- or infratentorial, in the spinal cord or the optic nerve. Patients with either clinical signs of activity (new relapse symptoms) or radiographic activity signs (new contrast-enhancing lesion) had a significantly higher CSF-CXCL13 value (*p* = 0.001 and *p* < 0.001, respectively; Figure 3a,b). In further correlation analyses (Table S4) for all 98 patients described above, leucocyte, lymphocyte, and plasma cell counts correlated with the level of CSF-CXCL13 (r = 0.538; r = 0.483 and r = 0.531, respectively). We observed no significant correlation for QAlb (r = 0.185) and the monocyte count (r = 0.021). Patients with CSF-specific oligoclonal IgG bands and intrathecal IgM synthesis had significantly higher CSF-CXCL13 values (*p* = 0.036 and *p* = 0.006). We thus demonstrated that CSF-CXCL13 is significantly increased in multiple sclerosis/ON/CIS/NMOSD patients with both clinical activity regarding new relapse symptoms and magnetic resonance imaging activity with new gadolinium-enhancing T1 lesions.

### 2.6. Differentiation of LNB from Other Neurological Disorders Using CSF-CXCL13–CSF-CXCL13 as a Diagnostic and Differentiation Marker

**Figure 4:**
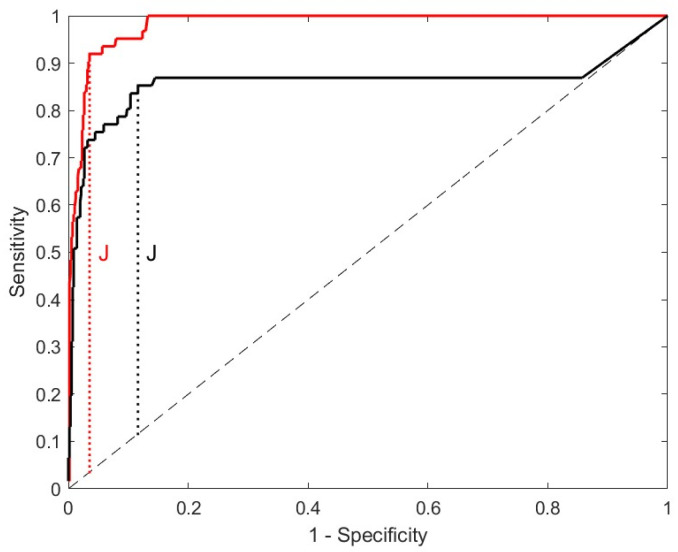
ROC curve analyses to determine a cut-off value for CSF-CXCL13 for neuroborreliosis; CXCL13 values are given in pg/mL (red curve) and ng/g total protein (black curve). Two receiver operating characteristic (ROC) curve analyses were performed (black and red curve) for different units of CXCL13 to obtain the optimal cut-off value for CSF-CXCL13 to distinguish neuroborreliosis from all other neuroinfectious and autoimmune (inflammatory) diseases, as well as non-inflammatory controls, with the highest sensitivity and specificity. The red curve represents the ROC analysis for CSF-CXCL13, in pg/mL. The second ROC analysis was performed for CSF-CXCL13, given as ng/g total protein to compensate for protein elevations due to blood–CSF barrier dysfunction. The cut-off after optimization using Youden’s index (J; black dotted line) under the same conditions was 428.92 in pg/mL (red curve) and 164.15 in ng/g total protein (black curve), respectively.

To investigate the role of CSF-CXCL13 as a (differential) diagnostic marker of neuroborreliosis, we performed receiver operating characteristic (ROC) curve analysis indicating the sensitivity and specificity of CSF-CXCL13 (Figure 4 and Figure S4, Table S5). In the ROC analysis, we included all 61 neuroborreliosis patients vs. all other patients from the NID, AIND, and NIND groups. We excluded patients with primary or secondary CNS lymphomas and solid brain tumors in the ROC analysis due to the lack of differential diagnostic potential [36]. The ROC analysis, followed by calculation of the Youden’s index, revealed the CSF-CXCL13 value of 428.92 pg/mL as the optimal cut-off with a sensitivity of 92.1% and specificity of 96.5% for the diagnosis of neuroborreliosis in our cohort (Figure 4).

In a further ROC analysis, using the same patient cohort, we calculated the cut-off of CSF-CXCL13 for the amount of CXCL13 in ng per gram of total protein (Figure 4) to compensate for protein differences due to the potential blood–CSF barrier dysfunction. Here, we obtained the best CSF-CXCL13 cut-off after applying Youden’s index at 164.15 ng/g total protein (sensitivity of 85.2% and specificity of 88.4% for the diagnosis of neuroborreliosis). Overall, sensitivity, and specificity were significantly lower than when using the amount of CSF-CXCL13 in pg/mL. In addition, the test performance, which is indicated by the area under the ROC curve (AUC), was significantly lower when calculating the cut-off in ng/g protein (0.98 vs. 0.86). A comparison of the ROC analyses of the two options for indicating the quantity of CSF-CXCL13 is given in Table 3.

In the non-inflammatory controls (NINDs), no patient exceeded the 428.92 pg/mL cut-off. The highest CSF-CXCL13 value in the control group was 51 pg/mL (idiopathic intracranial hypertension). Four patients with definite neuroborreliosis remained below the cut-off (142 pg/mL, 144 pg/mL, 243 pg/mL, and 333 pg/mL, respectively). All of these patients had peripheral facial nerve palsy, pleocytosis in the CSF (24–156/µL), an elevated albumin ratio (7.5–29.5), and intrathecal Bb-specific antibody synthesis (AI for IgG/IgM ≥ 1.5). We assumed neuroborreliosis because differential diagnoses were partially ruled out, and the symptoms and CSF parameters improved after intravenous ceftriaxone therapy. Three patients with cerebral cryptococcosis and concomitant neurosarcoidosis had CSF-CXCL13 levels considerably above the threshold (1380 pg/mL, 2307 pg/mL, 45,609 pg/mL), similar to in one patient with aspergillosis and CNS involvement (480 pg/mL). Three of them had meningoencephalitis, and one patient had meningitis due to cerebral cryptococcosis with vasculitis and stroke [17]. Similarly, three patients with optic neuritis due to serologically confirmed syphilis and probable neurosyphilis (Treponema pallidum specific AI < 1.5) but pleocytosis (3/3), blood–CSF barrier dysfunction (2/3), and intrathecal IgM synthesis (1/3) had CSF-CXCL13 levels surpassing the cut-off (5600 pg/mL, 1865 pg/mL, 959 pg/mL). One patient with streptococcal meningitis also had a significantly elevated value (720 pg/mL). Of the 185 patients with viral neurological infections and neuroinfectious diseases of unknown pathogens, only seven had CSF-CXCL13 levels above the cut-off. Of these, four patients had meningoencephalitis (one VZV, three unknown pathogens), one had polyradiculitis due to HSV-1, and two had myelitis and intracerebral abscess without pathogen detection. A patient with multiple sclerosis and acute relapse with multiple supra- and infratentorial and spinal gadolinium-enhancing T1 lesions in MRI had a CSF-CXCL13 value of 604 pg/mL. Likewise, two patients with CNS vasculitis had CSF-CXCL13 levels significantly above the 428.92 pg/mL cut-off. Of these, one patient (1347 pg/mL) had a rheumatologic cause with evidence of antinuclear Sm-antibodies. She had a headache, leptomeningeal contrast enhancement on MRI, and a blood sedimentation rate of 100 mm/h. One patient with paraneoplastic syndrome (sensomotoric polyneuropathy) due to small cell lung cancer with the detection of Zic4-antibodies had a CXCL13 value of 836 pg/mL, and lastly, a patient with the first diagnosis of Neuro-Behçet’s disease with di-mesencephalic-pontine lesions with contrast-enhancement in the T1 MRI-sequences had a CSF-CXCL13 level above the determined cut-off (CSF-CXCL13: 1150 pg/mL).

### 2.7. CSF-CXCL13 as a Possible Marker of Disease Severity to Differentiate an Uncomplicated from a Complicated Course in Neurological Infections of Different Origins

**Figure 5:**
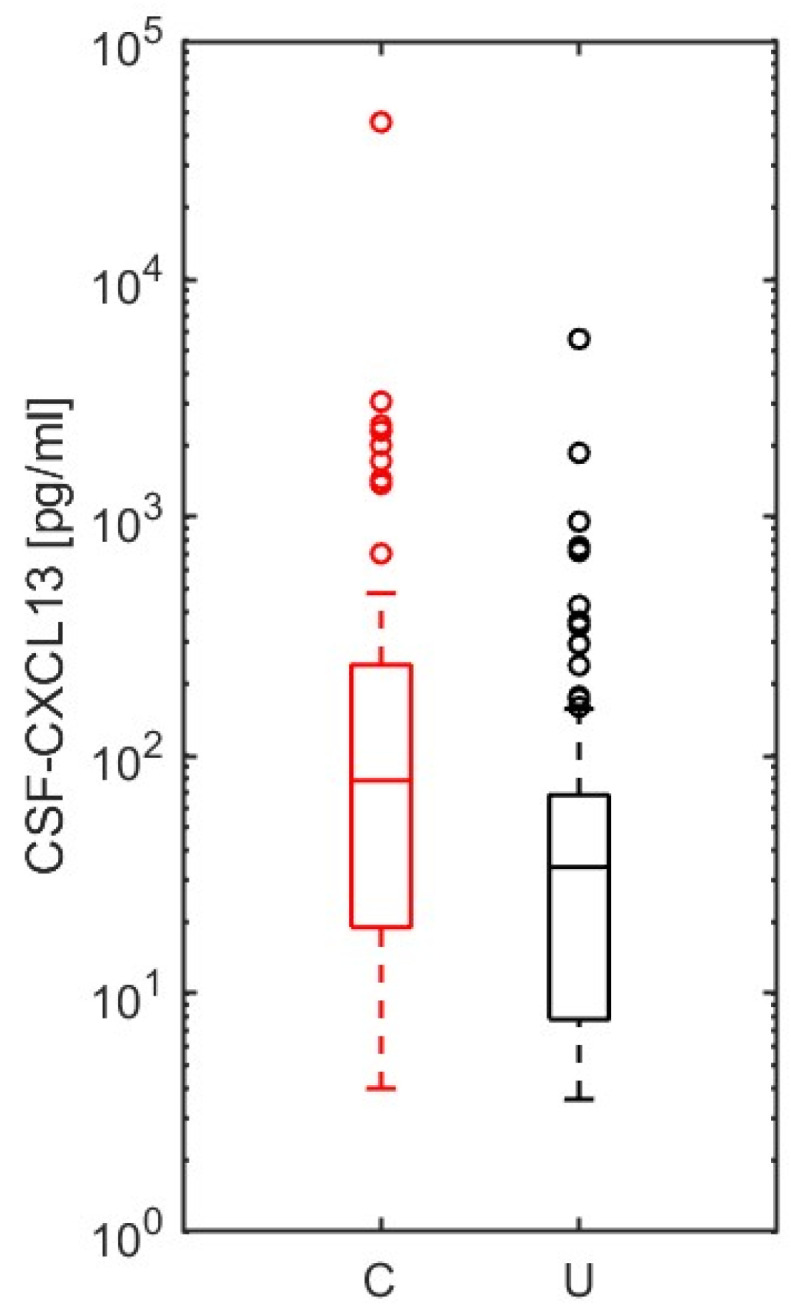
Evaluation of CSF-CXCL13 as a marker of disease severity in neuroinfectious diseases other than neuroborreliosis. Ninety-four complicated ((meningo-) encephalitis, myelitis, abscess) and 115 uncomplicated disease courses (meningitis, cranial nerve palsies, polyradiculitis) of neuroinfectious diseases were compared using the Mann–Whitney U test regarding CSF-CXCL13 (*p* < 0.001). *p*-values <0.05 were considered statistically significant. The values are represented as boxplots with interquartile ranges (bars), whiskers (dotted lines), medians (horizontal lines), and outliers (open circles).

Finally, we investigated the role of CSF-CXCL13 as a possible marker for a complicated course in neurological (CNS) infections. In the NID group, we dichotomized all patients, except neuroborreliosis cases, into complicated (with CNS-involvement; e.g., (meningo-) encephalitis, myelitis, abscess) and uncomplicated (without CNS-involvement; e.g., meningitis, peripheral facial nerve palsy, other cranial nerve palsies, polyradiculitis) courses similar to Pilz et al. [41]. Patients with complicated courses of disease (*n* = 94) had a significantly higher CSF-CXCL13 value (*p* < 0.001; Figure 5) than those with uncomplicated courses (*n* = 115). ROC curve analyses, which investigated the potential of CSF-CXCL13 as a possible marker to distinguish the different disease courses or to serve as a severity marker, were not successful. The test performance was poor (AUC = 0.34). A cut-off could not be determined after optimization based on Youden’s index (J = 0 for a cut-off 3.6 pg/mL, sensitivity 100%, specificity 0%). Thus, in our cohort, CSF-CXCL13 does not have the necessary performance to discriminate the course of neuroinflammatory disorders but might be a marker of disease severity.

## 3. Discussion

With this study, we aimed to clarify the diagnostic value and the potential of CSF-CXCL13 as a marker of activity and therapy of neuroborreliosis and beyond. Since CXCL13 was first described as a B-cell-attracting chemokine in 1998, there has been much debate as to whether it can serve as a biomarker in neuroborreliosis. However, CXCL13 is not only discussed as a potential diagnostic and treatment response biomarker in neuroborreliosis but also in primary CNS lymphoma and multiple sclerosis [19,21,44]. Its potential role as an activity marker has been under debate in the latter [44,45,46,47]. We set the target, especially for clinicians, to elaborate on the role of CSF-CXCL13 as a differential diagnostic biomarker of neuroborreliosis, an activity marker of autoimmune CNS diseases and a possible severity marker in neurological infectious CNS diseases. Thus, we would like to provide an overview of what clinicians can expect from determining CXCL13 in the CSF.

CSF-CXCL13 was previously considered mainly as a diagnostic biomarker of early neuroborreliosis [12]. Significant increases in CSF-CXCL13 were seen prior to intrathecal borrelia-specific antibody synthesis, as this can still be negative in the first six weeks in approximately 20% of neuroborreliosis cases [48,49]. In contrast, the antibody index can still be elevated (AI ≥ 1.5) years after neuroborreliosis and CSF-CXCL13 can, therefore, be used to distinguish between past/treated and active neuroborreliosis and might be a helpful marker indicating active infection and a need for therapy [49,50]. CSF-CXCL13 is the marker in neuroborreliosis that, unlike other viral and bacterial CNS infections, declines more sharply and rapidly than the leucocyte count, albumin quotient, and Bb-specific IgG AI [9]. In our cross-sectional study, CSF-CXCL13 was highly elevated in neuroborreliosis, cerebral cryptococcosis, neurosyphilis, aspergillosis with CNS involvement, and primary or secondary B-cell lymphoma, which is in line with previous studies [8,9,13,17,19,36,42,51]. At a 428.92 pg/mL cut-off, CSF-CXCL13 can distinguish neuroborreliosis from other neurological (CNS) autoimmune/inflammatory/infectious diseases with a sensitivity of 92.1% and specificity of 96.5%. In previous studies, ROC curve analyses have been performed to determine a cut-off for the differential diagnostic accuracy of CSF-CXCL13 in neuroborreliosis with a wide range of 18.9–1726 pg/mL [8,9,10,13,21,22,23,24,25,26,27,28,29,30,31,32,33,34,35,36,37,38]. One of these studies yielded a cut-off of 337 in ng/g total protein to compensate for potential protein differences and blood–CSF barrier dysfunction [9]. In our study, a cut-off in ng/g total protein was achieved at 164.15 with a considerably poorer sensitivity of 85.2% and specificity of 88.4% than when using pg/mL (under the same conditions) or compared with the study by Senel et al. (sensitivity 96.4%; specificity 96.9%) [9]. A significant reason for the divergence of the cut-offs in ng/g total protein may be the selection of the non-LNB group and the numerical ratio of LNB to the non-LNB group. Senel et al. included various differential diagnoses of neuroborreliosis. In contrast, our study compared various infectious or autoimmune CNS diseases and the non-inflammatory control group (many differential diagnoses of LNB) [9]. Therefore, based on our cohort, a cut-off of CSF-CXCL13 in pg/mL for discriminating neuroborreliosis seems to be more sensitive and specific.

We implemented the already established cut-offs (CXCL13 in pg/mL) in our cohort (Table S6 in Supplementary Materials). We found similar sensitivities of 88–100% in our patient cohort for cut-off values between 18.9 and 500 pg/mL. Specificity ranged from 56.89% (cut-off 18.9 pg/mL) to 95.94% and 96.64% (415 pg/mL and 500 pg/mL, respectively) [13,23]. For higher cut-offs (1229 pg/mL and 1726 pg/mL), a lower sensitivity (67.74% and 62.90%) but a higher specificity (97.88% and 98.59%) could be reached [8,24]. The reasons for the broad range of cut-offs are different. One possible explanation may be a distinct study population and the relationship between neuroborreliosis and the differential diagnoses. We excluded CNS-lymphoma due to the lack of differential diagnostic potential, similar to Hytönen et al. (cut-off 415 pg/mL) and van Burgel et al. (cut-off 215 pg/mL), but in contrast to Schmidt et al. (cut-off 1229 pg/mL) [8,13,36,42]. Two studies included neuroborreliosis patients pretreated with antibiotics [8,31]. The inter-center variability could also be due to various preanalytical conditions, such as different storage conditions prior to centrifugation (−20 °C vs. −80 °C), different processing times between lumbar puncture and further laboratory analysis (studies indicated a mean delay of up to 1.82 days), and different conditions during centrifugation, e.g., 1000× *g* and −50 °C vs. 2000× *g* and room temperature in our cohort [27,33,35]. Subsequent CSF analysis after up to five years of storage at −80 °C and up to four freeze-thaw cycles with serum does not seem to lead to a significant deviation in CXCL13 values. However, our samples were analyzed after six days at the latest [36,52]. In contrast, nine years of storage at −30 °C showed an inverse correlation between CXCL13 in CSF and the time of storage [8]. Other reasons for the variability in cut-offs could be the analytical tests used. The studies vary between conventional enzyme-linked immunosorbent assay (ELISA) kits (Euroimmun, Lübeck, Germany; Quantikine R&D Systems, Minneapolis, MN, USA) and bead-based assays (Mikrogen, Neuried, Germany; Merck Millipore, Burlington, MA, USA). Two studies comparing these analytic methods obtained different absolute values and cut-offs for the same study cohort, probably due to distinctly designed antibody pairs and interference with heterophilic antibodies [31,38].

In our cohort, the B-cell-attracting chemokine CXCL13 in CSF could not significantly distinguish different viral pathogen-related diseases, such as TBE, VZV-infections, or HSV-1 (meningo-) encephalitis from each other, or multiple sclerosis, limbic encephalitis, paraneoplastic syndrome, neuroinfectious diseases of unknown pathogens, or bacterial meningitis. Thus, as described earlier, CXCL13 appears to be the intrinsic response to specific lipoprotein surface structures, such as OspA and Pam3C in the spirochete species Borrelia burgdorferi and Treponema pallidum, in contrast to lipopolysaccharides of pneumococci [7]. In former studies, it could be shown that the value of CSF-CXCL13 is not dependent on the viral or bacterial species or autoantibodies in other pathogen-related and autoimmune CNS diseases [7,40,41,53]. However, this at least supports the differential diagnostic potential of the level of CSF-CXCL13 in distinguishing the two tick-borne diseases, TBE and neuroborreliosis, from each other, which, depending on the stage, can be similar in terms of symptoms, like (meningo-) encephalitis [54,55]. Our study shows that CSF-CXCL13 is significantly higher in neuroborreliosis than in TBE. As discussed above, the Bb-specific IgG or IgM AI may persist in the CSF for years after having had LNB, which may pose a differential diagnostic problem if lymphocytic pleocytosis is present at the same time, as may be the case with LNB and TBE [49,50,56,57]. In a previous study, the specificity of Bb-specific AI for diagnosing LNB was lower at 87.1% [9]. The level of CSF-CXCL13 could, therefore, serve as an important differential diagnostic tool if Bb- and TBE-specific AI are present in the same patient [58]. CSF-CXCL13 can distinguish LNB from other pathogen-related neurological diseases, such as TBE, with high sensitivity and specificity, from a cut-off of 428.92 pg/mL.

In previous studies, CXCL13 was >50-fold higher in CSF in primary and secondary CNS lymphomas than in non-inflammatory controls [42]. CXCL13 could be a homing factor for B lymphocytes before or after malignant transformation, migrating across the blood–CSF barrier into the CNS [19,42,51]. CSF-CXCL13 is discussed to be a helpful diagnostic biomarker together with CXCL9, β2-microglobulin, soluble interleukin-2-receptor, and interleukin-10 in CNS lymphoma [59,60,61]. In our cohort, the median of CSF-CXCL13 from primary and secondary B-cell lymphoma (826.3 pg/mL) and cerebral cryptococcosis (1843.5 pg/mL) were well above the determined cut-off for neuroborreliosis of 428.92 pg/mL. Likewise, no differentiation between these diseases was possible using the CSF-CXCL13 values. Little is known about cryptococcosis with CNS involvement and CSF-CXCL13 [17]. Three of the cases in our study had comorbidity with neurosarcoidosis without HIV. A comparative study described cryptococcosis in 28% of patients with neurosarcoidosis (cerebral cryptococcosis was described in 72%) [62]. The main risk factors for comorbidity and opportunistic infections were deficient cell-based immunity and immunosuppressive medication [62].

In addition, CSF-CXCL13 serves as a treatment response marker in various diseases. In contrast to other viral (e.g., TBE) or bacterial infections, CSF-CXCL13 levels in neuroborreliosis showed a significant decrease only a few days after the start of antibiotic therapy, in contrast to the leucocyte count, CSF/serum albumin ratio, or Bb-specific IgG AI [9,10,13,27,63]. According to a case report of a young female patient with cryptococcal meningitis, CSF-CXCL13 could also be a potential marker of the disease course and treatment response [17]. Similarly, in patients with CNS lymphoma who responded well to chemotherapy, a significant reduction in CSF-CXCL13 was observed compared to in patients with clinical and radiographic progression [19]. CSF-CXCL13 also correlated significantly with anti-NMDA receptor antibody production in limbic encephalitis with significantly lower CSF-CXCL13 levels in patients with favorable responses to first-line immunotherapies according to the modified Rankin Scale score [20].

The lymphoid chemokine CXCL13 serves mainly as a B-cell attractor to direct them into the B-cell follicles of secondary lymphoid organs (SLOs) or cerebrospinal fluid [64]. Data on correlation analyses of white blood cell counts and differential cytology are rather heterogeneous with a tendency for a positive correlation between CSF-CXCL13 and leucocyte, lymphocyte, and plasma cell counts in various viral and bacterial CNS infections, multiple sclerosis, neuroinfectious diseases of unknown pathogens, and primary CNS lymphomas, as shown in our study [4,9,10,13,14,15,19,24,26,31,40,44,45,46,47,53,65]. We also found a correlation with leucocyte and lymphocyte cell counts in our MS cohort, similar to former studies [14,15]. In patients with evidence of intrathecal IgM synthesis or CSF-specific oligoclonal IgG bands, CSF-CXCL13 was significantly higher in neuroinfectious diseases and multiple sclerosis patients in our study, in line with results from previous studies [15,44,45,65]. Therefore, CXCL13 is not only restricted to the acute phase of inflammation, but as seen in the example of tertiary lymphoid structures in MS or neuroinfectious diseases, it also seems to be involved in chronic inflammation and the humoral phase of infection.

The mainly intrathecal-produced chemokine CXCL13 showed no correlation with the CSF/serum albumin ratio (QAlb) in non-inflammatory controls and multiple sclerosis patients as described before [9,15,48,65]. However, a correlation between CSF-CXCL13 and QAlb could be found in neuroborreliosis or other neuroinfectious diseases. One possible explanation may be a higher intensity of inflammation and, thus, increased meningeal involvement with additional leakage of CXCL13 from the blood. In contrast, Pilz et al. discuss the exclusively intrathecal production of CSF-CXCL13 [66].

Two sites of CXCL13 production are discussed in MS patients. In relapsing-remitting MS patients, immunohistochemical and quantitative PCR analyses have detected increased levels of CXCL13 in perivascular spaces and the extracellular matrix in early and highly active MS lesions characterized by high numbers of CD68+ macrophages [15]. In these patients, CXCL13 was also strongly dependent on intrathecal IgG production, B cells, and T cells in the CSF, whereas no CXCL13 could be detected in chronically inactive lesions [15]. Follicle-like structures in the meninges (tertiary lymphoid organs; TLOs), which resemble germinal centers of B-cell follicles but without structured organization, are thought to be the site of CXCL13 production in progressive MS [67]. The TLOs were described in approximately 30–40% of patients with secondary progressive MS in autopsy studies [68]. It is still unclear whether TLOs exist during the acute course of the disease or are formed only during chronification [67]. Additionally, in patients who developed RRMS after CIS or optic neuritis, CSF-CXCL13 was significantly higher [45,65,69,70]. Multiple sclerosis/CIS patients with clinical or radiographic relapse symptoms, gadolinium-enhancing T1 lesions, or enlarging T2 lesions in MRI had elevated CSF-CXCL13 levels compared to those in patients with primary or secondary progressive MS (PPMS/SPMS) [44,45,46,47]. Intrathecally produced CXCL13 tends to be an activity marker in MS in general [71]. In our study, MS patients with relapse and new contrast-enhancing T1 lesions on MRI, including the brain and spinal cord, also had higher CXCL13 values in the CSF. CSF-CXCL13 correlated with inflammatory activity in the CSF (leucocyte count, lymphocytes, CSF-specific oligoclonal IgG bands, intrathecal IgM synthesis) in our MS cohort as well.

Compared to neuroborreliosis, data on other infectious or autoimmune neurological diseases are relatively rare, especially regarding the prognostic value of CSF-CXCL13 and its role as a severity marker. For neuroinfectious diseases, these are mainly based on two pilot studies with a small population of aseptic/purulent (meningo-) encephalitis (*n* = 12 and *n* = 26, respectively) [40,41]. Fujimori et al. established a cut-off at 197.84 pg/mL that distinguishes aseptic meningoencephalitis from meningitis with a sensitivity and specificity of 100% each [40]. The number of patients was deficient, with four meningoencephalitis cases and eight meningitis cases [40]. Similarly, in a slightly larger cohort (*n* = 26), Pilz et al. also showed a higher CSF-CXCL13 value, as well as CSF/serum albumin ratio, in the first and second lumbar puncture in complicated courses (*n* = 8; encephalitis +/− vasculitis, myelitis) in contrast to meningitis/monoradiculitis (uncomplicated course; *n* = 18) [41]. In this cohort, a 250 pg/mL cut-off could discriminate between the two courses with 100% sensitivity and 83% specificity [41]. In our larger cohort (*n* = 209) of neuroinfectious diseases other than neuroborreliosis, we found a significantly higher CSF-CXCL13 value in the encephalitis/myelitis/abscess group (complicated course; *n* = 94), but no cut-off could be established through ROC curve analysis. CSF-CXCL13 is thus higher in these complicated neuroinfectious diseases, but the diagnostic performance in differentiation from uncomplicated courses (*n* = 115; meningitis, cranial nerve palsies, polyradiculitis) is too weak regarding only CSF-CXCL13 values. The median time from symptom onset to the first lumbar puncture was approximately the same: 7 days in our cohort for neuroinfectious diseases vs. 8.5 days (complicated course) and four days (uncomplicated course) in Pilz et al. and 6.5 days (complicated course) in Fujimori et al., respectively [40,41]. In summary, although CXCL13 is higher in complicated CNS inflammation and, therefore, might be a marker of the severity of the infection; the diagnostic accuracy is insufficient to distinguish between complicated and uncomplicated disease courses. Further analysis is needed to show which routine parameters increase the diagnostic performance and prognostic value of CSF-CXCL13.

Besides molecular biomarkers, there is an expanding field of genetic and epigenetic biomarkers or predisposing factors, especially in autoimmune and cancer diseases, as discussed [72,73]. One of the most famous genetic susceptibility factors is HLA-B51 for Behçet’s disease [74]. There is also evidence that deficiencies in epigenetics are associated with disease worsening [75]. Most deficiencies include deficits in DNA methylation due to dysfunctional enzymes [76]. DNA epigenetic modifications also include the silencing of virus genomes via methylation after an acute infection [76]. Perhaps persisting virus genomes due to epigenetic deficits could explain why encephalitis patients experience complicated diseases instead of meningitis patients. A combination with CXCL13 would be interesting and maybe enhance its biomarker potential for disease severity.

### Strengths and Limitations

One of the main strengths of our study is the large number of patients with complete CSF and serum findings and clinical records using an electronic database. In addition, the cross-sectional retrospective design also represents a strength. Over an extended period, patients who received a complete analysis of CSF, including CXCL13, were collected, and by cross-sectioning, we thus obtained a vast spectrum of diseases. Besides Lintner et al., this is the most extensive study concerning CSF-CXCL13 to date [29]. We have also included a vast range of differential diagnoses of LNB and neurological disorders. The study is intended to reflect real-life clinical conditions. The laboratory method for measuring CXCL13 (ELISA) is widely used and remained the same over the entire period under review.

The retrospective design as a strength also represents one of the study’s limitations. Due to the study’s retrospective nature, follow-up lumbar punctures were not performed systematically and only in some patients. Such studies with a systematic investigation are rare and of small size. The timing of the lumbar puncture after symptom onset also varied. However, according to our analyses, this is not easy to systematize in everyday clinical practice and does not seem to have any correlation,. Furthermore, these are pre-selected patients from the hospital’s Biobank and do not represent the entire patient cohort of the hospital who received lumbar punctures from July 2009 to January 2023 with measurements of CXCL13 in CSF. The retrospective design implicates further biases, such as the various subgroups differing in the size, gender ratio, and age. However, age and gender do not play a role concerning the level of CSF-CXCL13, as we and others could demonstrate [14,65].

## 4. Materials and Methods

### 4.1. Patients and Selection Process

**Figure 6:**
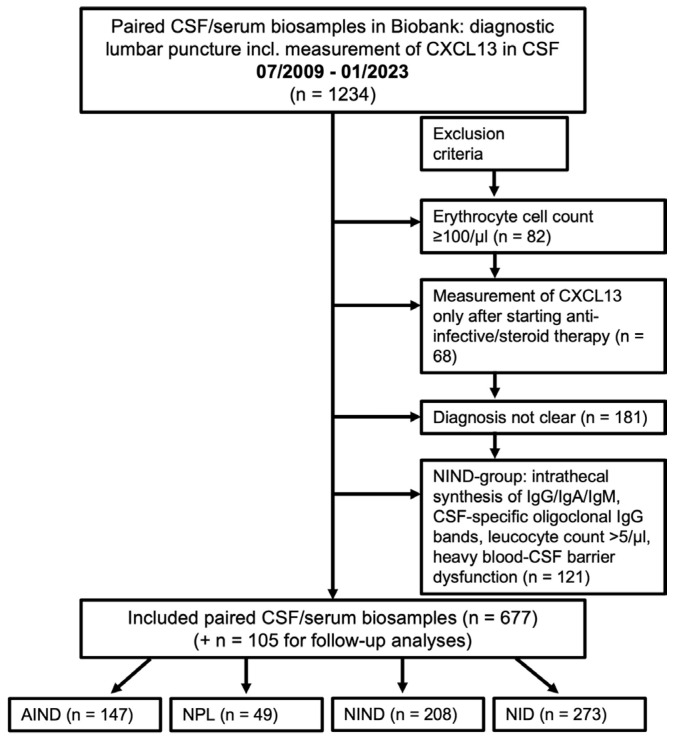
Patient selection process. The patient selection process is described in this flowchart. From July 2009 to January 2023, 1234 patients of the Biobank of the Department of Neurology, University Hospital of Ulm, Germany, received a lumbar puncture, including the measurement of CXCL13. According to the above criteria, 677 patients were selected and divided into the 4 groups for further analysis. CXCL13: C-X-C-motif chemokine ligand 13, CSF: cerebrospinal fluid, AIND: autoimmune inflammatory neurological diseases, NPL: CNS-neoplasia, NIND: non-inflammatory neurological diseases, NID: neuroinfectious diseases.

We retrospectively included paired CSF/serum samples from all patients of the Biobank (patients who agreed to the scientific use of their medical data and biosamples) of the Department of Neurology, University Hospital Ulm, Germany, who received a CSF analysis including measurements of the CSF-CXCL13 value from July 2009 to January 2023 (*n* = 1234) as part of a diagnostic lumbar puncture. The patients, their diseases, and data of the CSF and blood analysis were identified based on the electronic database of the University Hospital Ulm. By reviewing the medical charts, we obtained findings on CSF leucocyte counts, cell differentiation, lactate, the blood–CSF barrier function (CSF/serum albumin ratio), intrathecal IgG, IgM, IgA synthesis, and CSF-specific oligoclonal IgG bands. Patients whose medical history was not well-documented or whose discharge diagnosis was missing were excluded (*n* = 181). Patients with either an erythrocyte count ≥100/µL in the CSF analysis (*n* = 82; possible false positive CSF-lactate or CSF-albumin) or measurements of CSF-CXCL13 values only after starting anti-infective therapy or corticosteroids (*n* = 68) were also excluded. However, the follow-up lumbar punctures of the neuroborreliosis and other neuroinfectious disease patients were used for further analyses (105 samples). In our control group, acute or chronic inflammatory CNS disease was excluded (inclusion criteria: leucocyte cell count ≤5/µL, CSF-specific oligoclonal IgG bands negative, no evidence of intrathecal IgG, IgA, or IgM synthesis, no/mild/moderate dysfunction of the blood–CSF barrier according to the CSF/serum albumin ratio). Therefore, a further 121 patients were excluded. The resulting 677 patients were divided into four groups: non-inflammatory neurological diseases (NIND; *n* = 208), autoimmune (inflammatory) neurological diseases (AIND; *n* = 147), neuroinfectious diseases (NID; *n* = 273), and CNS-neoplasia (NPL; *n* = 49). The NIND group served as a control group. Table S1 in Supplementary Materials section lists the included diseases. For the group of AIND, we included multiple sclerosis (MS; *n* = 73; 61 with relapsing-remitting type, 6 with the secondary progressive type, and 6 with the primary progressive type according to the 2017 revised criteria of McDonald [77]), clinically isolated syndrome (CIS; *n* = 3), isolated optic neuritis (ON; *n* = 18), neuromyelitis optic spectrum disease (NMOSD; *n* = 4), autoimmune (limbic) encephalitis with antibody detection [78,79] (*n* = 14), paraneoplastic syndrome other than limbic encephalitis [78,80] (*n* = 11), chronic lymphocytic inflammation with pontine perivascular enhancement responsive to steroids (CLIPPERS) (*n* = 1), a neurological manifestation of Sjögren’s syndrome (*n* = 1), CNS-vasculitis (*n* = 8), Neuro-Behçet’s disease [81,82] (NBD; *n* = 3), and neurosarcoidosis (*n* = 10). Of the 73 MS patients, 65 were not receiving immunomodulatory or immunosuppressive treatment at the time of lumbar puncture, as well as two CIS and two NMOSD patients. The remaining patients, including their therapy, were distributed as follows: glatiramer acetate (2 MS, 1 CIS), azathioprine (1 MS), interferon beta 1-alpha (1 MS, 1 NMOSD patient who was considered to have multiple sclerosis at the time of the lumbar puncture), ocrelizumab (1 MS), fingolimod (2 MS), alemtuzumab (1 MS), and nilotinib (1 NMOSD with chronic myelogenous leukemia). Two patients with Neuro-Behçet’s disease had already received immunosuppressive therapy (mycophenolate mofetil, ciclosporin). In the NID group, we included patients with clinical signs of meningitis, encephalitis, myelitis, cranial nerve palsies, polyradiculitis, or a combination thereof. Encephalitis was defined according to the major and minor criteria of the International Encephalitis Consortium [79]. In the CSF analyses, they had pleocytosis (leucocyte cell count > 5/µL), blood–CSF barrier dysfunction (QAlb), and corresponding pathogen detection or not as described below. A viral, bacterial, fungal, or parasitic etiology was confirmed via polymerase chain reaction (PCR), using cultural/microscopic evidence, or by determining pathogen-specific antibodies in CSF/serum pairs and calculating the antibody indices (AIs). In patients with neurosyphilis, we further added the Treponema pallidum particle agglutination/hemagglutination (TPPA/TPHA) test in serum and CSF, the Venereal Disease Research Laboratory (VDRL) test in CSF, and the fluorescent treponemal antibody absorption (FTA-ABS) test in serum according to Klein et al. [83]. The following diseases were included in the NID group: Lyme neuroborreliosis (LNB; *n* = 61; all definite cases according to Stanek et al. [84]), bacterial meningitis (*n* = 7), neurosyphilis (*n* = 10, of which two patients had human immunodeficiency virus (HIV), aspergillosis (*n* = 2), neuro schistosomiasis (*n* = 1), cryptococcosis (*n* = 4; including three patients with additional neurosarcoidosis, one patient with HIV), varicella-zoster virus infection (VZV; *n* = 49), herpes simplex virus type 1 infection (HSV-1, *n* = 12), Epstein–Barr virus disease (EBV; *n* = 2), or tick-borne meningoencephalitis (TBE; *n* = 24). One patient in the cryptococcosis cohort has already been described in a case report [17]. To the NID group, we also added neuroinfectious diseases of unknown pathogens (I-UPs; *n* = 101). These also included meningitis, encephalitis, myelitis, radiculitis, or cranial nerve palsies with the previously described CSF findings but without successful pathogen detection in the investigations, as mentioned earlier. However, based on the CSF analysis and laboratory and clinical findings, the most likely origin appears to be viral or atypical pathogens. The group of CNS-neoplasia included patients with an initial diagnosis of primary or secondary CNS B- or T-cell non-Hodgkin’s lymphoma or solid brain tumors confirmed through brain biopsy and/or CSF immunophenotyping via flow cytometry. These included diffuse large B-cell lymphoma (*n* = 14), plasmocytoma (*n* = 5), follicular B-cell lymphoma (*n* = 2), extranodal marginal zone lymphoma (*n* = 1), mycosis fungoides (*n* = 1), primary intestinal epitheliotropic T-cell lymphoma (*n* = 1), B-cell chronic lymphocytic leukemia (*n* = 2), astrocytoma WHO grade I/II/III (*n* = 3), glioblastoma multiforme (*n* = 12), meningioma (*n* = 3), oligodendroglioma WHO grade II (*n* = 1), and ganglioglioma WHO grade 1 (*n* = 1). No histology was obtained in three cases. An overview of the selection process and exclusion criteria can also be taken from Figure 6. Table S1 in Supplementary Materials section includes a further survey of the four superordinate groups with the assigned diagnoses.

### 4.2. Laboratory CSF Analyses

After the diagnostic lumbar puncture, the samples were handled adhering to the guidelines of the German Society for Cerebrospinal Fluid Diagnostics and Clinical Neurochemistry (DGLN) and the German Society of Neurology (DGN) [85]. The collection, processing, and storage of samples were under international standards for biomarker research in Biobanks [86]. Analyses of the leucocyte count (cells/µL), cell differentiation (% of leucocytes), total protein (mg/L), age-related CSF/serum albumin concentration ratio (QAlb) as an assessment of blood–CSF barrier integrity (age-dependent cut-off for QAlb < (4 + age/15) × 10^−03^), lactate (mmol/L), immunoglobulins A/G/M (IgA/G/M), and CSF-specific oligoclonal IgG bands were performed as previously described [85,87,88,89,90]. Leucocytes were counted manually using a Fuchs–Rosenthal hemocytometer. In this cell counting chamber, quantitative differential cytology was also carried out manually under the microscope, and monocytes and plasma cells were given as a percentage of the leucocyte count. The quantitative intrathecal IgG, IgA, and IgM expression and the intrathecally produced fraction (%) were calculated using CSF/serum quotients of the corresponding immunoglobulins (QIgG, QIgA, QIgM) concerning the upper limits of their reference ranges [88]. We used an enzyme-linked immunosorbent assay (ELISA) according to the manufacturer’s instructions (Gold Standard Diagnostics Europe (former Genzyme Virotech), Rüsselsheim, Germany) for the detection of antibody levels against varicella-zoster virus, herpes simplex virus type 1, measles virus, rubella virus, Borrelia burgdorferi, tick-borne encephalitis virus, and Epstein–Barr virus. In order to investigate the intrathecal synthesis of the antibodies just determined, the following formula for the calculation of a specific antibody index (AI) was applied: AI = QIg[spec]/QIg[total] for QIg[total] < Qlim(Ig) (upper reference limit for QIg) and QIg[spec]/Qlim(Ig) for QIg[total] > Qlim(Ig) [90]. We determined Qlim(Ig) using Reiber’s formula [88]. We considered AI levels ≥1.5 indicative of the intrathecal immunoglobulin synthesis of the investigated antigen [90]. The value of CXCL13 in CSF and serum was also determined through an ELISA according to the manufacturer’s instructions (until 01/2018 Quantikine R&D Systems, Minneapolis, MN, USA; since 02/2018 Euroimmun, Lübeck, Germany). We performed a method comparison of the two manufacturers of the CXCL13-ELISA. As specified by the manufacturer Euroimmun, we confirmed concordant results with a correlation coefficient of 0.98 (corresponding to R^2^ = 0.97) in 40 patients. The assay range specified by the manufacturer was 7.8–500 pg/mL for Quantikine, R&D Systems and 4.6 pg/mL for Euroimmun. For statistical analysis, the results “<7.8 pg/mL” and “<4.0 pg/mL” were set to 7.8 pg/mL and 4.0 pg/mL, respectively, as described earlier [21,24]. CSF-CXCL13 values <10 pg/mL were considered normal. The calculation of concentrations higher than the highest detection level was achieved based on appropriate dilutions and re-analyzation. CXCL13 was analyzed after ≤6 days. Until then, samples centrifuged at 2000 × *g* for 10 min were stored at +2 to +8 °C. Testing for intracellular and surface autoantibodies was performed by performing line-blot and cell-based assays, as well as indirect immunofluorescence on unfixed sections of the brain, nerve, pancreas, and intestine of primates (all Euroimmun, Lübeck, Germany) in CSF and serum. Included antibodies were against Amphiphysin, CV2 (CRMP5), PNMA2 (Ma-2/Ta), Ri, Yo, Hu, recoverin, SOX1, titin, Zic4, GAD65, Tr (DNER), glutamate-receptor type (NMDA, AMPA1/2), contactin-associated protein 2 (CASPR2), leucine-rich glioma-inactivated protein 1 (LGI1), GABAB1 receptor, and DPPX.

### 4.3. Ethics

Written informed consent was obtained from all patients in accordance with the Declaration of Helsinki to collect biosamples (especially CSF and blood) upon lumbar and venipuncture in our Biobank at the Neurological University hospital of Ulm (ethics approval number 20/10 by the local ethics committee of the University of Ulm). This retrospective study was separately approved by the same ethics committee (approval number 242/23).

### 4.4. Statistics

All statistical analyses, including graphics, were performed with MATLAB R2022b (2022, The Mathworks^®^ Inc., Natick, MA, USA) for Microsoft^®^ Windows 11 Pro (2021, Redmond, WA, USA) and Microsoft^®^ 365 Excel for Mac (2021, Redmond, WA, USA). Testing for normal distribution was performed using the Kolmogorov–Smirnov test. Neither the age, CSF-CXCL13 value, leucocyte cell count, total protein, nor QAlb was normally distributed. Therefore, the median, interquartile range (25th to 75th percentile), and range were used to describe these continuous variables. The absolute and relative frequencies were calculated for discrete variables (gender, CSF-specific oligoclonal IgG bands, and intrathecal IgM synthesis). Group differences were calculated using the nonparametric methods: the Kruskal–Wallis test for continuous variables (more than two unpaired groups) and Pearson’s chi-squared test for discrete variables. Statistical significance was determined for *p*-values < 0.05. Further analyses were performed using the Mann–Whitney U test (difference between two unpaired groups) and the Dunn’s post hoc test (multiple comparisons for >2 groups), respectively [91]. We used the Wilcoxon signed-rank test for a comparison of two paired groups. Correlations were determined using Spearman’s rank coefficient (ρ, further “r”). Receiver operating characteristic (ROC) curve analysis was performed to investigate the accuracy of CSF-CXCL13 in distinguishing neuroborreliosis from other neurological autoimmune and infectious diseases. The same analysis was performed to investigate how accurately CSF-CXCL13 can distinguish an uncomplicated course (meningitis/peripheral cranial nerve palsy) from a complicated course (encephalitis/meningoencephalitis/myelitis) in the neuroinfectious diseases (NID) group. The area under the ROC curve (AUC) was also determined as a measurement of the performance of the ROC analysis [92]. Youden’s index ((sensitivity + specificity) − 1) was calculated for each cut-off value to determine which cut-off increases the accuracy (highest Youden’s index) and maximizes sensitivity and specificity [93].

## 5. Conclusions and Future Perspective

**Figure 7:**
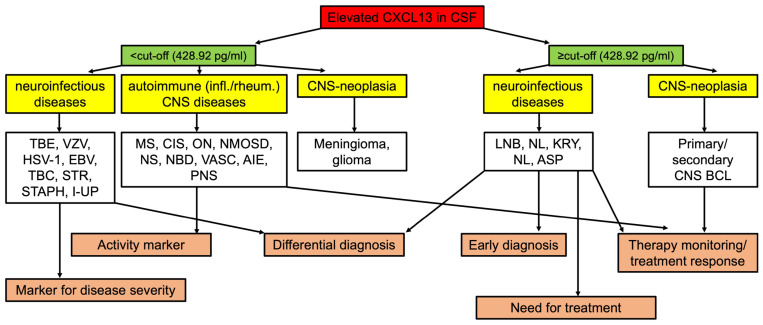
Flowchart for elevated CSF-CXCL13. This flowchart represents an overview for clinicians of how they could integrate elevated CSF-CXCL13 in their clinical routine for various neurological disorders based on our patient cohort. EBV: Epstein–Barr virus, MS: multiple sclerosis, I-UP: neuroinfectious diseases of unknown pathogen, NBD: Neuro-Behçet’s disease, TBE: tick-borne meningoencephalitis, STR: streptococcus, STAPH: staphylococcus, VZV: varicella zoster virus, CIS: clinically isolated syndrome, VASC: CNS-vasculitis, AIE: autoimmune encephalitis, NMOSD: neuromyelitis optica spectrum disease, NS: neurosarcoidosis, PNS: paraneoplastic syndrome (other than limbic encephalitis), HSV-1: herpes simplex virus-1, TBC: tuberculosis, NL: neurosyphilis, BCL: B-cell lymphoma, KRY: cryptococcosis, LNB: Lyme neuroborreliosis, ON: optic neuritis.

In our retrospective study, we emphasized the role of CSF-CXCL13 as a diagnostic and treatment response marker, primarily in neuroborreliosis, and as an activity marker in multiple sclerosis and related diseases. Many scenarios highlight its benefits for clinicians. (A) As described above, CSF-CXCL13 could be used as a marker that indicates the re-infection of neuroborreliosis and differentiates the need for treatment. (B) In neuroinfectious diseases, CSF-CXCL13 can be used for differential diagnosis since in some diseases, such as neuroborreliosis, cryptococcal meningitis, or neurosyphilis, very high CSF-CXCL13 values could be observed. (C) Additionally, as described in our study and the literature, CSF-CXCL13 can be used for therapy monitoring in neuroborreliosis, cryptococcal meningitis, autoimmune encephalitis, and primary CNS B-cell lymphoma [9,10,17,19,20]. As summarized in Table 4 and Figure 7, implementing CXCL13 into the clinical routine diagnostics is discussed considering its advantages and limitations and the proposed cut-offs.

CSF-CXCL13 is used to differentiate neuroborreliosis from other neurological conditions but cannot discriminate viral, other bacterial, autoimmune, and neuroinfectious diseases of unknown pathogens. In approximately 60% of viral encephalitis and up to 40% of encephalitis cases, no causative agent or pathogen can be detected [94,95]. In a future analysis, other components, e.g., the complement system, could be investigated as a further link between the innate and adaptive immune systems to achieve an etiological classification of the meningitis/encephalitis of the unknown pathogens group. Furthermore, the role of CSF-CXCL13 in patients with the exclusion of acute CNS inflammation, but those with evidence of chronic CNS inflammation of an unknown cause, could be further investigated. We could only include manual differential cytology in routine diagnostics within the retrospective design for correlation analyses of leucocyte subpopulations. A further prospective study could extend this to immunophenotyping and correlation analyses of lymphocyte subpopulations, natural killer cells, monocytes, and macrophages with CSF-CXCL13. CSF-CXCL13 is an activity marker in multiple sclerosis patients, so the added value compared to MRI findings warrants further investigation.

## Figures and Tables

**Table 1:** Demographic characteristics and CSF findings.

	NIND	NID	AIND	NPL	*p*-Value
*n* (female/male)	208 (91/117) (43.8%/46.2%)	273 (99/174) (36.3%/63.7%)	147 (87/60) (59.2%/40.8%)	49 (21/28) (42.3%/57.7%)	<0.001 **
Age (years), median (range)	54 (3–93)	56 (14–91)	45 (4–88)	66 (34–86)	<0.001 *
Leucocyte count (/µL), median (range)	1 (0–5)	101 (6–480)	7 (0–54)	3 (0–31)	<0.001 *
Total protein (mg/L), median (range)	491 (136–1470)	957 (179–3268)	555 (165–1670)	874 (189–2610)	<0.001 *
QAlb (CSF/S), median (range)	6.3 × 10^−3^ (1.9 × 10^−3^–14.7 × 10^−3^)	12.5 × 10^−3^ (2.4 × 10^−3^–34.6 × 10^−3^)	6.2 × 10^−3^ (2.5 × 10^−3^–16.2 × 10^−3^)	9.3 × 10^−3^ (3.1 × 10^−3^–26.6 × 10^−3^)	<0.001 *
CSF-CXCL13 (pg/mL), median (range)	7.8 (4–13)	76 (4–1466)	32 (4–224)	43 (4–2120)	<0.001 *
CSF-specific oligoclonal IgG bands; *n* (+/−), (+)	0/208 (0)	117/156 (42.9%)	116/31 (78.4%)	4/45 (8.2%)	<0.001 **
Intrathecal IgM synthesis; *n* (+/−), (+)	0/208 (0)	88/185 (32.2%)	30/117 (20.3%)	5/44 (10.2%)	<0.001 **

Differences in the four groups were determined using a Kruskal–Wallis test * for continuous variables (age, leucocyte count, total protein, QAlb, CSF-CXCL13) and Pearson’s chi-squared test ** for discrete variables (gender, oligoclonal IgG bands in CSF, intrathecal IgM synthesis). All *p*-values are rounded. Values for continuous variables are given as the median, and range and values for discrete variables are given as absolute and relative counts. QAlb: CSF-albumin/serum-albumin (albumin ratio), CSF: cerebrospinal fluid, AIND: autoimmune inflammatory neurological diseases, NPL: CNS-neoplasia, NIND: non-inflammatory neurological diseases; NID: neuroinfectious diseases.

**Table 2:** Correlation analyses of CSF-CXCL13 and different routine CSF findings in the four groups.

Parameter	NIND	NID	AIND	NPL
*n*	208	273	147	49
Leucocyte count (/µL)	r = 0.045	r = 0.132	r = 0.560	r = 0.655
Lymphocyte count (/µL)	r = 0.158	r = 0.139	r = 0.495	r = 0.748
Monocyte count (/µL)	r = 0.120	r = 0.066	r = 0.163	r = 0.358
Plasma cell count (/µL)	-	r = 0.271	r = 0.311	-
QAlb (CSF/S)	r = 0.135	r = 0.347	r = 0.336	r = 0.674

Correlation analyses between CSF-CXCL13 and the routine CSF parameters were performed using Spearman’s rank coefficient (r). Correlation analyses of plasma cells in NIND and NPL could not be performed due to the low number of detected cells with differential cytology. All r-values are rounded. QAlb: CSF-albumin/serum-albumin (albumin ratio), CSF: cerebrospinal fluid, AIND: autoimmune inflammatory neurological diseases, NPL: CNS-neoplasia, NIND: non-inflammatory neurological diseases, NID: neuroinfectious diseases.

**Table 3:** Parameters of different ROC curve analyses for neuroborreliosis using different units for CSF-CXCL13.

Unit	Cut-Off	AUC	Youden’s Index	Sensitivity	Specificity
pg/mL	428.92	0.98	0.88	92.1%	96.5%
ng/g total protein	164.15	0.86	0.74	85.2%	88.4%

ROC curve analyses were performed to determine a cut-off for CSF-CXCL13 for neuroborreliosis with two different units for CSF-CXCL13 using the same patient cohort. We used “pg/mL” and “ng/g total protein” to compensate for elevations in protein due to possible blood–CSF barrier dysfunction. Youden’s index was calculated to obtain the optimal cut-off of each ROC analysis. The AUC represents the area under the ROC curve and indicates test performance. The calculated values for the cut-off of CSF-CXCL13, AUC, Youden’s index, sensitivity, and specificity are shown here. ROC: receiver operating characteristic, AUC: area under the curve.

**Table 4:** Advantages and limitations of CSF-CXCL13 in different neurological disorders.

Neurological Disorder/Disease	Advantages	Limitations
Neuroborreliosis (LNB)	- Marker of very early LNB (before measurement of Bb-specific AI) [49,50] - Discriminative marker between past and acute infection (indicates an active infection and need for therapy) [49,50] - High diagnostic accuracy at a cut-off of 428.92 pg/mL (sensitivity 92.1%, specificity 96.5%) - Marker of treatment response	- High elevation also in possible differential diagnoses (cryptococcus meningitis, neurosyphilis, aspergillosis with CNS involvement)
Neuroinfectious diseases (other than LNB)	- Marker of disease severity - Useful marker for differential diagnosis of infections of unknown origin	- No differentiation between various pathogens
MS/ON/CIS/NMOSD	- Marker of clinical (acute relapse) and radiographic (new contrast-enhancing T1 lesions) disease activity	- No differentiation between autoimmune CNS disease and acute neuroinfectious disease
Autoimmune (limbic) encephalitis/paraneoplastic syndrome	- Marker of treatment response [20] - Clinical activity marker [20]	- No differentiation between autoimmune CNS disease and acute neuroinfectious disease
Primary/secondary B-cell CNS lymphoma	- Marker of treatment response [19]	- Very high CSF-CXCL13 values (similar to LNB)

MS: multiple sclerosis, ON: optic neuritis, CIS: clinically isolated syndrome, NMOSD: neuromyelitis optica spectrum disease.

## Data Availability

The data that support the findings of this study are available anonymized on reasonable request from the corresponding author. The data are not publicly available due to privacy.

## Supplementary Materials

The following supporting information can be downloaded at: https://www.mdpi.com/article/10.3390/ijms25010425/s1.
