# Supplementary material for: CXCL13 in Cerebrospinal Fluid: Clinical Value in a Large Cross-Sectional Study
Source: Int J Mol Sci. 2023 Dec 28;25(1):425. doi: 10.3390/ijms25010425 (PMC10779058; doi:10.3390/ijms25010425)
Supplement: Supplementary file 1 [file ijms-25-00425-s001.zip › ijms-2766800-supplementary.pdf]

**Table S1. Classification of different diseases into the 4 groups AIND, NID, NPL, and NIND.**

**Autoimmune (inflammatory/rheumatic) neurological diseases (AIND; n = 147)**

- Multiple sclerosis (n = 73)
  - Relapsing-remitting type (n = 61)
  - Secondary progressive type (n = 6)
  - Primary progressive type (n = 6)
- Clinically isolated syndrome (n = 3)
- Isolated optic neuritis (n = 18)
- Neuromyelitis optica spectrum disease (n = 4)
  - Aquaporin-4-antibody positive (n = 3)
  - Antibody-negative (n = 1)
- Autoimmune (limbic) encephalitis (n = 14)
- Paraneoplastic syndrome (other than limbic encephalitis) (n = 11)
  - Sensory neuronopathy (n = 5)
  - Sensorimotor neuropathy (n = 1)
  - Cerebellar degeneration (n = 6)
- CLIPPERS (n = 1)
- Sjögren’s syndrome (neurological) (n = 1)
- CNS-vasculitis (n = 8)
  - Primary angiitis of the central nervous system (PACNS) (n = 4)
  - Parainfectious in case of hepatitis C (n = 1)
  - Unknown origin (n = 2)
  - Rheumatic with anti-nuclear antibodies (Sm) (n = 1)
- Neuro-Behçet’s disease (n = 3)
  - Parenchymal type (n = 2)
  - Non-parenchymal type (n = 1)
- Neurosarcoidosis (n = 10)

**Neuroinfectious diseases (NID; n = 273)**

Bacterial (n = 79)

- Neuroborreliosis (n = 61)
  - Definitive (n = 61)
- Bacterial meningitis (n = 7)
  - Staphylococcus aureus (n = 2)
  - Streptococcus pneumoniae (n = 2)
  - Staphylococcus epidermidis (n = 1)
  - Streptococcus agalactiae (n = 1)
  - Mycobacterium tuberculosis (n = 1)
- Neurosyphilis (n = 10)
  - Definitive (n = 5)
  - Probable (n = 5)
    - Incl. HIV (n = 2)

Fungal/parasitic (n = 7)

- Aspergillosis with CNS-involvement (n = 2)
- Neuroschistosomiasis (n = 1)
- Cryptococcosis with CNS-involvement (n = 4)
  - Incl. neurosarcoidosis (n = 3)
  - Incl. HIV (n = 1)

Viral (n = 87)

- Varicella-zoster virus (n = 49)
- Herpes-simplex virus type 1 (n = 12)
- Epstein-Barr virus (n = 2)
- Tick-borne meningoencephalitis virus (n = 24)

Unknown pathogen (n = 101)

- Neuroinfectious diseases of unknown pathogen (n = 101)

| |
|---|
| **CNS-neoplasia (NPL; n = 49)**<br>B-cell non-Hodgkin lymphoma (n = 21; Primary CNS lymphoma (n = 16); Secondary CNS lymphoma (n = 9))<br>– Diffuse large cell B-cell lymphoma (n = 14)<br>– Waldenstrom's macroglobulinaemia (n = 5)<br>– Follicular B-cell lymphoma (n = 2)<br>– Marginal zone lymphoma (n = 1)<br>– B-cell chronic lymphocytic leukemia (n = 2)<br>– No histology (n = 2)<br>T-cell non-Hodgkin lymphoma (n = 2; Secondary CNS lymphoma (n = 2))<br>– Cutaneous T-cell lymphoma (mycosis fungoides; n = 1)<br>– Primary intestinal epitheliotropic T-cell lymphoma (n = 1)<br>Solid brain tumor (n = 21)<br>– Meningioma (n = 3)<br>– Pilocytic astrocytoma (n = 1)<br>– Anaplastic astrocytoma (n = 1)<br>– Diffuse astrocytoma (n = 1)<br>– Glioblastoma (n = 12)<br>– Oligodendroglioma (WHO-grade II; n = 1)<br>– Ganglioglioma (n = 1)<br>– No histology (n = 1) |
| **Non-inflammatory neurological diseases (NIND; n = 208)**<br>Primary headache disorders (n = 19), idiopathic intracranial hypertension (n = 3), rheumatological diseases without CNS involvement (n = 11), Parkinsons disease (n = 3), somatoform disorders (n = 17), non-inflammatory sensomotoric polyneuropathy (n = 16), restless legs syndrome (n = 1), epilepsy/first epileptic seizure (n = 23), stroke/transitory ischemic attack (n = 13), myasthenia gravis (n = 3), amyotrophic lateral sclerosis (n = 10), Huntington's chorea (n = 3), myositis/myalgia (n = 5), Bell's palsy (n = 12), transitory global amnesia (n = 1), Alzheimer's disease (n = 2), vascular dementia (n = 4), primary progressive aphasia (n = 1), lumbago/lumboischialgia (n = 11), spinal canal stenosis (n = 2), myelo-dysplastic syndrome (n = 2), cranial nerve palsies (n = 6), Phelan McDermid syndrome (n = 8), fatigue syndrome (n = 10), psychiatric diseases (n = 4), systemic borreliosis (n = 3), dural arteriovenous fistula (n = 1), hypokalemic periodic paralysis (n = 1), neuralgic shoulder amyotrophy (n = 3), |

| |
|---|
| normal pressure hydrocephalus (n = 1), spinal muscular atrophy type 1/3 (n = 3), stiff-person syndrome (n = 3), multiple system atrophy (n = 2), giant cell arteritis (n = 1) |

Patients of the Biobank of the Department of Neurology, University Hospital of Ulm, who had received a lumbar puncture from July 2009 to January 2023 were categorized into the 4 groups using medical chart review.

**Table S2. Differences in routine CSF findings and CSF-CXCL13 in the four groups.**

| **Parameter** | **NIND-NID** | **NIND-AIND** | **NIND-NPL** | **NID-AIND** | **NID-NPL** | **AIND-NPL** |
|---|---|---|---|---|---|---|
| CSF-CXCL13 | **p < 0.001** | **p < 0.001** | **p < 0.001** | **p < 0.001** | **p = 0.275** | **p = 0.134** |
| Leucocyte cell count | **p < 0.001** | **p < 0.001** | **p < 0.001** | **p < 0.001** | **p < 0.001** | **p = 0.043**<br>p > 0.05* |
| QAlb | **p < 0.001** | p = 0.631 | **p < 0.001** | **p < 0.001** | **p = 0.024**<br>p > 0.05* | **p = 0.001** |
| Total protein | **p < 0.001** | p = 0.105 | **p = 0.008** | **p < 0.001** | p = 0.133 | p = 0.156 |

Mann-Whitney U test and the more precise Dunn's post hoc test* were used for multiple comparisons between the 4 groups. P-values below 0.05 were considered significant according to Dunn's test. All values are rounded. QAlb: CSF-albumin/serum-albumin (albumin ratio), CSF: cerebrospinal fluid, AIND: autoimmune inflammatory neurological diseases, NPL: CNS-neoplasia, NIND: non-inflammatory neurological diseases; NID: neuroinfectious diseases.

**Figure S1. Analyses of CSF-CXCL13 and gender or age in the NIND group.**

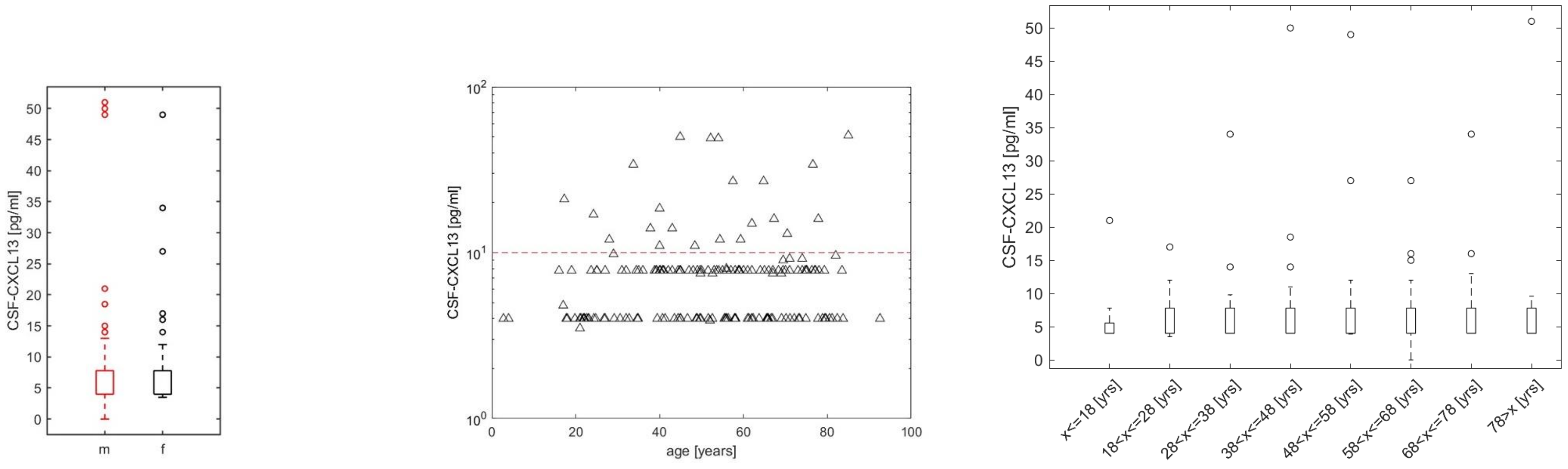


a) b) c)

**Figure S1. a)** The patients of the NIND group were divided according to gender (m: masculine and f: feminine). The Mann-Whitney U test was used to compare the values of CSF-CXCL13 in the two groups. No significant difference was detected (p = 0.384). **b)** Correlation analyses between CSF-CXCL13 and age in the NIND group were determined using Spearman's rank coefficient (r). The dotted lines represent the cut-off for CSF-CXCL13 for non-inflammatory controls (<10 pg/ml) in red. No significant correlation was obtained for CSF-CXCL13 and age (r = 0.078). **c)** The patients of the NIND group were grouped by age in steps of ten (yrs: years). Patients below the age of 18 and above 78 were summarized. The Kruskal-Wallis test was used to compare the values of CSF-CXCL13 in the different age-groups. No significant difference was found (p = 0.156).

**Figure S2. CSF-CXCL13 value and CSF-specific oligoclonal IgG bands and intrathecal IgM synthesis**

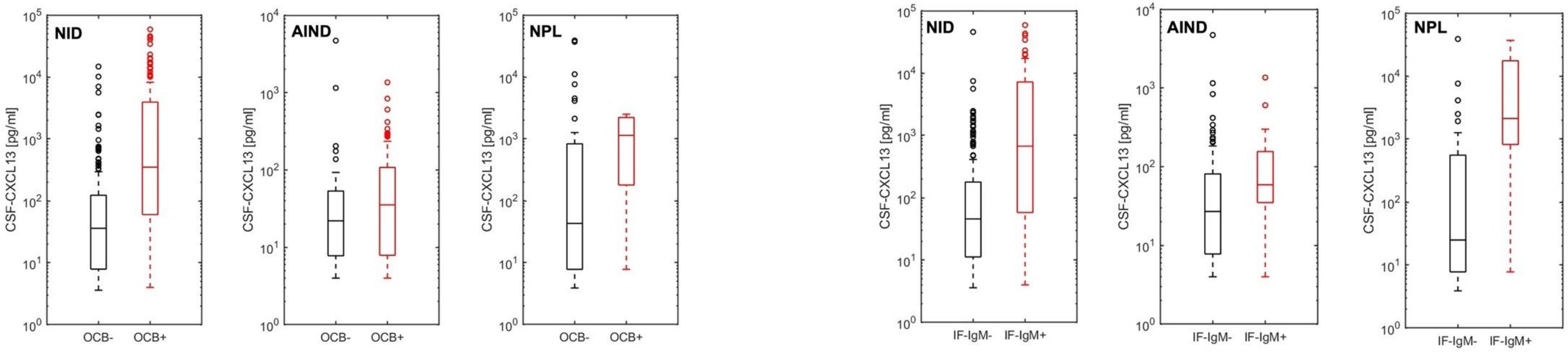


a) b)

**Figure S2.** The patients of the three different groups NID, AIND, and NPL were grouped **a)** according to CSF-specific oligoclonal IgG bands (OCB+: OCB present and OCB-: OCB absent) and **b)** intrathecal IgM synthesis (IF-IgM+: intrathecal IgM synthesis present and IF-IgM-: intrathecal IgM synthesis absent). The Mann-Whitney U test was applied for possible significant differences regarding CSF-CXCL13 between each positive and negative status. Patients of the NID group with CSF-specific oligoclonal IgG bands and all groups with intrathecal IgM synthesis had significantly higher CSF-CXCL13 values. **a)** NID: $p < 0.001$, AIND: $p = 0.273$, NPL: $p = 0.307$ and **b)** NID: $p < 0.001$, AIND: $p = 0.001$, NPL: $p = 0.034$. NID: neuroinfectious diseases, AIND: autoimmune (inflammatory) diseases, NPL: CNS-neoplasia, IF-IgM: intrathecal fraction of IgM (=intrathecal IgM synthesis), OCB: CSF-specific oligoclonal IgG bands.

**Figure S3. Classification of MS/CIS/ON/NMOSD patients according to clinical and radiographic activity and location of the lesions with contrast enhancement in MRI.**

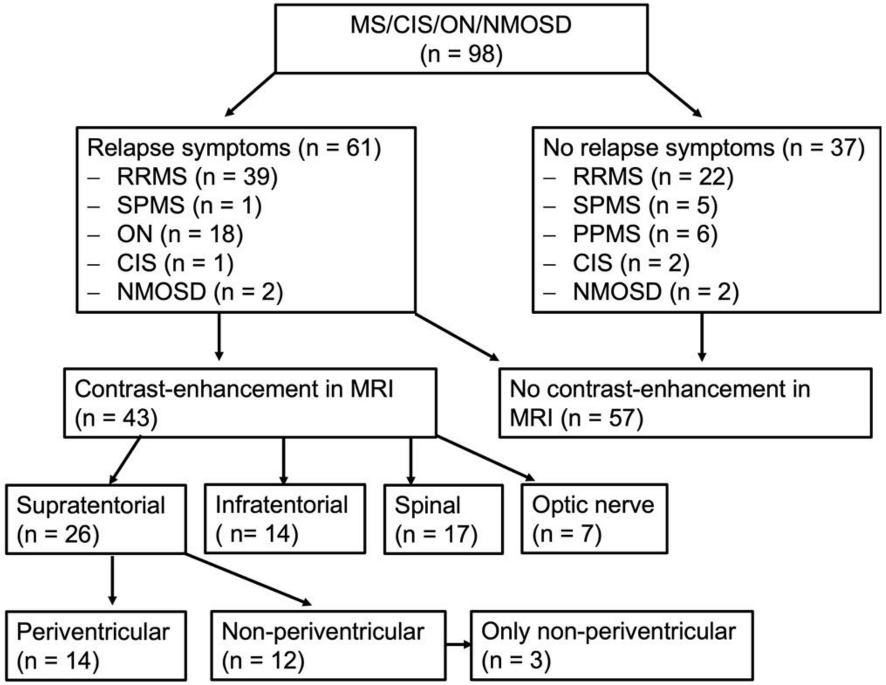


**Figure S3.** Ninety-eight patients with multiple sclerosis (MS), clinically isolated syndrome (CIS), optic neuritis (ON), and neuromyelitis optica spectrum disease (NMOSD) were grouped according to clinical and radiographic activity by location.

**Table S3. Calculation of CSF/serum ratios and indices for CXCL13 in various neurological conditions.**

| **Disease** | **S-CXCL13 median (range) [pg/ml]** | **CSF-CXCL13 median (range) [pg/ml]** | **QAlb median (range)** | **Q-CXCL13 = CSF-CXCL13/S-CXCL13 median (range)** | **I-CXCL13 = QCXCL13/ QAlb median (range)** |
|---|---|---|---|---|---|
| LNB | 73.0 (21 - 304) | 1932 (142 - 17200) | $13.6 \times 10^{-3}$ ($3.0 \times 10^{-3}$ - $36.2 \times 10^{-3}$) | 24.08 (0.08 - 148.32) | 1747.03 (7.9 - 12864.4) |
| BCL | 50.1 (28 - 217) | 695 (7.8 - 2500) | $12.4 \times 10^{-3}$ ($5.6 \times 10^{-3}$ - $127.1 \times 10^{-3}$) | 8.36 (0.11 - 24.35) | 734.6 (6.16 - 3676) |
| Viral/I-UP/F/P/B-CNS-D | 63 (7.8 - 160) | 36.5 (4 - 227) | $11.8 \times 10^{-3}$ ($4.4 \times 10^{-3}$ - $28.4 \times 10^{-3}$) | 0.51 (0.03 - 3.22) | 47.96 (0.5 - 254.68) |
| MS/ON/CIS/NMOSD; all | 59.5 (7.8 - 158) | 23.5 (7.8 - 119) | $5.46 \times 10^{-3}$ ($2.51 \times 10^{-3}$ - $12.7 \times 10^{-3}$) | 0.39 (0.03 - 1.73) | 67.65 (4.82 - 255.02) |
| MS/ON/CIS/NMOSD; intact B-CSF-B | 61 (7.8 - 108) | 17.5 (7.8 - 97) | $5.27 \times 10^{-3}$ ($2.51 \times 10^{-3}$ - $8.8 \times 10^{-3}$) | 0.28 (0.03 - 1.43) | 67.65 (4.82 - 295.11) |
| AIE/PNS | 75 (21 - 137) | 65 (7.8 - 209) | $8.8 \times 10^{-3}$ ($3.44 \times 10^{-3}$ - $20.8 \times 10^{-3}$) | 0.72 (0.17 - 5.77) | 98.63 (9.33 - 359.48) |
| NIND | 64 (7.8 - 191) | 7.8 (-) | $6.8 \times 10^{-3}$ ($2.87 \times 10^{-3}$ - $15.3 \times 10^{-3}$) | 0.13 (0.008 - 0.33) | 18.9 (1.48 - 83.01) |

The ratio of CSF-CXCL13 and serum-CXCL13 (Q-CXCL13) was formed to calculate the CXCL13 index (I-CXCL13) in a further step using QAlb. Values are indicated as medians and range. QAlb: CSF-albumin/serum-albumin (albumin ratio); LNB: Lyme neuroborreliosis, BCL: primary/secondary CNS B cell lymphoma, I-UP: neuroinfectious diseases of unknown pathogens, F: fungal, P: parasitic, B-CNS-D: bacterial CNS disease, MS: multiple sclerosis, ON: optic neuritis, CIS: clinically isolated syndrome, NMOSD:

neuromyelitis optica spectrum disease, B-CSF-B: blood CSF barrier, AIE: autoimmune encephalitis, PNS: paraneoplastic syndrome, NIND: non-inflammatory neurological disease.

**Figure S4. ROC curve analyses to determine a cut-off value for CSF-CXCL13, Q-CXCL13, and I-CXCL13 for neuroborreliosis.**

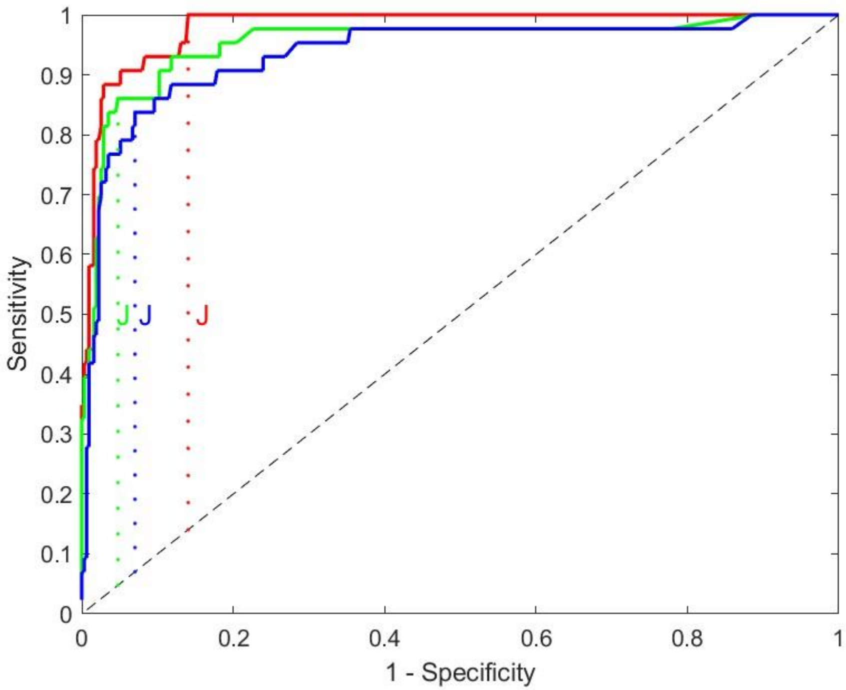


**Figure S4.** After calculation of a CSF-CXCL13/serum-CXCL13 ratio (Q-CXCL13) and an index (I-CXCL13 = Q-CXCL13/QAlb,) we performed receiver operating characteristic (ROC) curve analysis for CSF-CXCL13 (red curve), Q-CXCL13 (green curve), and I-CXCL13 (blue curve). We wanted to obtain the optimal cut-off value for these by using the Youden’s index to distinguish neuroborreliosis from all other neuroinfectious and autoimmune (inflammatory) diseases as well as non-inflammatory controls with the highest sensitivity and specificity. We also wanted to investigate if CSF-CXCL13 could be used without regarding the corresponding serum value for calculating

ratio and index, respectively. We only had serum-CXCL13 values from 315 patients of the non-LNB group and from 43 LNB-patients. The cut-off after optimization using Youden's index (J; black dotted line) was 126.88 pg/ml for CSF-CXCL13 (red curve), 5.73 for Q-CXCL13 (green curve), and 0.36 for I-CXCL13 (blue curve). Sensitivity, specificity, the area under the curve (AUC), and the Youden's index are indicated in Table S5.

**Table S4. Correlation analyses of CXCL13 and routine CSF findings in neuroborreliosis and MS/ON/CIS/NMOSD.**

| **Parameter** | **Neuroborreliosis (Spearman's r; p-value)** | **MS/ON/CIS/NMOSD (Spearman's r; p-value)** |
|---|---|---|
| Leucocyte count [/µl] | r = 0.426 | r = 0.538 |
| Lymphocyte count [/µl] | r = 0.471 | r = 0.483 |
| Monocyte count [/µl] | r = 0.329 | r = 0.021 |
| Plasma cell count [/µl] | r = 0.508 | r = 0.531 |
| QAlb (CSF/S) | r = 0.446 | r = 0.185 |
| CSF-specific oligoclonal IgG bands (+) | p = 0.022* | p = 0.036* |
| Intrathecal IgM synthesis (+) | p < 0.001* | p = 0.006* |

Correlation analyses between CXCL13 and the routine CSF parameters were determined using Spearman's rank coefficient (r) in neuroborreliosis and MS/ON/CIS/NMOSD. Mann-Whitney U test* was used to compare CSF-CXCL13 levels if CSF-specific oligoclonal IgG bands or intrathecal IgM synthesis were present or absent. All values are rounded and values <0.05 were considered significant. QAlb: CSF-albumin/serum-albumin (albumin ratio), CSF: cerebrospinal fluid.

**Table S5. Parameters of different ROC-analyses for neuroborreliosis using CSF-CXCL13, Q-CXCL13, and I-CXCL13.**

| | **Cut-off** | **Sensitivity** | **Specificity** | **AUC** | **Youden‘s index** |
|---|---|---|---|---|---|
| CSF-CXCL13 [pg/ml] | 127 | 100% | 85.94% | 0.98 | 0.86 |
| CSF-CXCL13/serum-CXCL13 (= Q-CXCL13) | 5.73 | 86.05% | 95.21% | 0.95 | 0.81 |
| Q-CXCL13/QAlb (= I-CXCL13) | 0.36 | 83.72% | 92.97% | 0.94 | 0.77 |

After calculation of a CSF-CXCL13/serum-CXCL13 ratio (Q-CXCL13) and an index (I-CXCL13 = Q-CXCL13/QAlb), we performed receiver operating characteristic (ROC) curve analysis for each (see Figure S4). Calculated cut-offs, sensitivity, specificity, the area under the ROC curve (AUC), and the Youden’s index are shown here. The area under the ROC curve (AUC) indicating the test performance was highest for CSF-CXCL13 (0.98). Therefore, calculations with CSF-CXCL13 alone might be sufficient.
We only considered patients with measurement of CSF-CXCL13 and serum-CXCL13 in these ROC-analyses (n = 315 for the non-LNB group and n = 43 for LNB-patients).

**Table S6. CSF-CXCL13 cut-offs for neuroborreliosis from the literature implemented in our patient cohort.**

| Reference [no.] | Total individual count (n) | LNB count for ROC-analysis (n) def./prob. | Non-LNB cohort | Method, manufacturer | Proposed cut-off [pg/ml], [ng/g total protein]* | Sensitivity (≙ TPR) [%]; for our data set | Specificity (≙ 1-FPR) [%]; for our data set | Youden‘s index (J); for our data set |
|---|---|---|---|---|---|---|---|---|
| Hytönen et al., 2014 [13] | 366 | 38 (def. + prob.) | Viral CNS-inf., NL, MS, non-infl. co. | ELISA, Quantikine | 415 | 91.94 | 95.94 | 0.88 |
| Schmidt et al., 2011 [8] | 205 | 27 (def.) | Viral/bacterial/fungal CNS-inf. (M/E), MS, prim./sec. CNS-lymphoma, non-infl. co. | ELISA, Quantikine | 1229 | 67.74 | 97.88 | 0.66 |
| van Burgel et al., 2011 [36] | 268 | 58 (def. + prob.) | Viral/bacterial/fungal CNS-inf., MS/ADEM, HIV, non-infl. co. | ELISA, Quantikine; ELISA, Quantikine | 250 | 93.55 | 92.40 | 0.86 |
| Wagner et al., 2018 [10] | 459 | 20 (def.) | Viral/bacterial CNS-inf., MS, stroke, limbic encephalitis, non-infl. co., intracranial malignancies/TU | ELISA, Euroimmun | 93.83 | 100.00 | 82.51 | 0.83 |
| Markowicz et al., 2018 [31] | 100 | 25 (def.) | TBE, aseptic M/ME (undefined pathogen) | recomBead CXCL13, Mikrogen; ELISA, Euroimmun | 131<br>259 | 100.00<br>93.65 | 86.22<br>92.52 | 0.86<br>0.86 |

| | | | | | | | | |
|---|---|---|---|---|---|---|---|---|
| Rupprecht et al., 2018 [17] | Meta-analysis (18 studies; 2944 individuals) | - | - | ELISA, Quantikine; ELISA, Euroimmun | 91 (cross-sectional studies)<br>164 (case-control studies) | 100.00<br>95.16 | 82.12<br>87.63 | 0.82<br>0.83 |
| Remy et al., 2017 [21] | 185 | 53 (def.) | Viral CNS-inf., non-infl. co. | ELISA, Quantikine | 55 | 100.00 | 75.09 | 0.75 |
| Henningsson et al., 2018 [26] | 191 | No ROC-analysis | Viral CNS-inf., stroke, non-infl. co. | recomBead CXCL13, Mikrogen | 160 | 96.77 | 87.63 | 0.84 |
| Cerar et al., 2013 [23] | 174 | 46 (def. + prob.) | TBE, MS, non-infl. co. | ELISA, Quantikine | 18.9 | 100.00 | 56.89 | 0.57 |
| Tjernberg et al., 2011 [35] | 261 | 124 (def.) | Head/neck pain, CNP, radiculitis | ELISA, Quantikine | 142 | 100.00 | 86.75 | 0.87 |
| Wutte et al., 2011 [37] | 75 | No ROC-analysis | Viral meningitis, CNP, systemic borreliosis, non-infl. co. | ELISA, Quantikine | 0<br>500 | 100.00<br>88.71 | 0<br>96.64 | 0<br>0.85 |
| Ljøstadt et al., 2008 [30] | 116 | No ROC-analysis | Viral/bacterial CNS-inf. (M/E), MS, non-infl. co. | ELISA, Quantikine | 125<br>500 | 100.00<br>88.71 | 85.87<br>96.64 | 0.86<br>0.85 |
| Knudtzen et al., 2020 [27] | 619 | 37 (def.) | Viral/bacterial CNS-inf. (M/E), NL, NS, cer. malignancies incl. prim. CNS-lymphoma, non-infl. co. | ELISA, Euroimmun | 49 | 100.00 | 71.02 | 0.71 |

| | | | | | | | | |
|---|---|---|---|---|---|---|---|---|
| Henningsson et al., 2016 [38] | 132 | 35 (def.) | Non-infl. co. | ELISA, Quantikine; recomBead CXCL13, Mikrogen; | 56<br>158 | 100.00<br>96.77 | 75.27<br>87.63 | 0.75<br>0.84 |
| Lintner et al., 2020 [29] | 1410 | 29 (def.) | Viral/bacterial/fungal CNS-inf., MS, intracranial malignancies/TU, non-infl. co. | ELISA, Euroimmun | 55.5 | 100.00 | 75.27 | 0.75 |
| Pìcha et al., 2016 [33] | 244 | 74 (def. + prob.) | Aseptic CNS-inf., Bell‘s palsy, peripheral neuropathy, non-infl. co. | ELISA, Quantikine | 29 | 100.00 | 62.01 | 0.62 |
| Barstadt et al., 2020 [22] | 195 | 77 (def. + prob.) | Aseptic meningitis/encephalitis, non-infl. co. | Multiplex bead assay, Millipore corp. | 213 | 95.16 | 90.63 | 0.86 |
| Eckman et al., 2021 [24] | 162 | 19 (def. + prob.) | Viral/fungal CNS-inf. (M/E), NL, KRY, MS, non-infl. co. | Multiplex bead assay, Thermo Fisher Sci. | 1726 | 62.90 | 98.59 | 0.61 |
| van Gorkom et al., 2021 [25] | 156 | 15 (def. + prob.) | Viral/bacterial/fungal CNS-inf., TBC, NL, MS, non-infl. co. | ELISA, Quantikine; recomBead CXCL13, Mikrogen | 85.9<br>252.2 | 100.00<br>93.55 | 80.74<br>92.40 | 0.81<br>0.86 |

| | | | | | | | | |
|---|---|---|---|---|---|---|---|---|
| Leth et al., 2022 [28] | 130 | 31 (def.) | Viral/bacterial CNS-inf., non-infl. co. | Bead-based assay, Bio-Rad | 50.7 | 100.00 | 72.44 | 0.72 |
| Senel et al., 2010 [9] | 126 | 28 (def.) | Viral/bacterial CNS-inf. (M/E), non-infl. co. | ELISA, Quantikine | 337* | 77.05 | 93.57 | 0.71 |

The cut-offs from the literature, either determined by ROC analyses or set as in-house cut-off, were implemented in our data set and sensitivity (TPR) and specificity (1-FPR) including Youden's index J were calculated (last three columns of the table). One cut-off (proposed by Senel et al., 2010 [9]) is given as ng/g total protein to compensate for differences in CSF protein. For the implementation of this cut-off in our cohort, the CSF-CXCL13 values were also given in ng/g total protein. Def.: definitive, prob.: probable, TPR: true positivity rate, FPR: false positivity rate, MS: multiple sclerosis, NL: neurosyphilis, TBC: tuberculosis, M/E: meningitis/encephalitis, non-infl. co: non-inflammatory controls, ADEM: acute demyelinating encephalomyelitis, HIV: human immunodeficiency virus, TU: tumor, KRY: cryptococcosis, TBE: tick-borne meningoencephalitis, CNP: cranial nerve palsies.
